# International Veterinary Epilepsy Task Force’s current understanding of idiopathic epilepsy of genetic or suspected genetic origin in purebred dogs

**DOI:** 10.1186/s12917-015-0463-0

**Published:** 2015-08-28

**Authors:** Velia-Isabel Hülsmeyer, Andrea Fischer, Paul J.J. Mandigers, Luisa DeRisio, Mette Berendt, Clare Rusbridge, Sofie F.M. Bhatti, Akos Pakozdy, Edward E. Patterson, Simon Platt, Rowena M.A. Packer, Holger A. Volk

**Affiliations:** Centre for Clinical Veterinary Medicine, Ludwig-Maximilians-University, Veterinärstr. 13, 80539 Munich, Germany; Department of Clinical Sciences of Companion Animals, Utrecht University, Yalelaan 108, 3583 CM, Utrecht, The Netherlands; Animal Health Trust, Lanwades Park, Kentford, Newmarket, CB8 7UU Suffolk, United Kingdom; Department of Veterinary and Clinical Sciences, Faculty of Health and Medical Sciences, University of Copenhagen, Frederiksberg C, Denmark; Fitzpatrick Referrals, Halfway Lane, Eashing, Godalming, GU7 2QQ Surrey, United Kingdom; School of Veterinary Medicine, Faculty of Health & Medical Sciences, University of Surrey, Guildford, GU2 7TE Surrey, United Kingdom; Department of Small Animal Medicine and Clinical Biology, Faculty of Veterinary Medicine, Ghent University, Salisburylaan 133, Merelbeke, 9820 Belgium; Clinical Unit of Internal Medicine Small Animals, University of Veterinary Medicine, Veterinärplatz 1, 1210 Vienna, Austria; University of Minnesota College of Veterinary Medicine, D426 Veterinary Medical Center, 1352 Boyd Avenue, St. Paul, MN 55108 USA; College of Veterinary Medicine, University of Georgia, 501 DW Brooks Drive, Athens, GA 30602 USA; Department of Clinical Science and Services, Royal Veterinary College, Hatfield, AL9 7TA Hertfordshire UK

**Keywords:** Idiopathic epilepsy, Dog, Breed, Epilepsy prevalence, Epileptic seizure

## Abstract

Canine idiopathic epilepsy is a common neurological disease affecting both purebred and crossbred dogs. Various breed-specific cohort, epidemiological and genetic studies have been conducted to date, which all improved our knowledge and general understanding of canine idiopathic epilepsy, and in particular our knowledge of those breeds studied. However, these studies also frequently revealed differences between the investigated breeds with respect to clinical features, inheritance and prevalence rates. Awareness and observation of breed-specific differences is important for successful management of the dog with epilepsy in everyday clinical practice and furthermore may promote canine epilepsy research. The following manuscript reviews the evidence available for breeds which have been identified as being predisposed to idiopathic epilepsy with a proven or suspected genetic background, and highlights different breed specific clinical features (e.g. age at onset, sex, seizure type), treatment response, prevalence rates and proposed inheritance reported in the literature. In addition, certain breed-specific diseases that may act as potential differentials for idiopathic epilepsy are highlighted.

## Introduction

Canine idiopathic epilepsy is a common neurological disease and has been recently defined as two or more unprovoked seizures at least 24 h apart with no identifiable underlying etiology other than a suspected genetic origin. Idiopathic epilepsy still represents a diagnosis of exclusion and an appropriate diagnostic workup is essential as a correct diagnosis impacts treatment and breeding decisions [1]. Affected dogs most often require life-long antiepileptic medication and regular control visits. Consequently, the daily lives of many owners are affected by concerns related to their pet’s seizures and the changes in daily routine [1–4]. Furthermore, canine idiopathic epilepsy is a disease, which is characterised by a broad array of clinical signs, age of onset, and at least in part underlying genetic backgrounds (see also Tables 1 and 2) [5, 6]. In recent years, idiopathic epilepsy with a proven or suspected genetic background has been reported for a number of purebred dogs with most studies focusing on clinical characteristics and genetic aspects. However, most studies have not yet identified causative gene mutations, suggesting that, either the research group in question did not have resources or availability to go from clinical to genetic identification of monogenic epilepsy-causing genes or that inheritance may be complex, involving several or many susceptibility genes, and be reflective of additional environmental interactions similar to what is proposed for many human genetic epilepsies [5–7]. The individual dog's response to antiepileptic treatment may also be complex and in some individuals, successful antiepileptic drug treatment presents a time- and cost-consuming challenge with an increased risk for poor quality of life, premature death or euthanasia when seizures cannot be adequately controlled [8–13]. Estimated prevalence data among the general dog population have been reported with variable results [14–17]. The true prevalence of epilepsy in dogs is unknown and has been estimated to be 0.6–0.75 % in general dog population [16, 18]. However, prevalence rates may differ considerably when looking at hospital populations with prevalence rates of 0.5 − 5 % in non-referral population and of 1–2.6 % in referral hospital population [14–17, 19–22]. In breeds, which are predisposed to idiopathic epilepsy, considerable higher prevalence rates are reported [23–26] than the prevalence estimated for the general dog population (see Table 3.), which is one of the reasons a genetic component is suspected in certain canine breeds. Current data show that the clinical courses, seizure semiology, treatment responses and heritability may differ substantially between dog breeds and also between geographically distinct populations of the same breed, which further highlights the complexity of the disease. In summary, knowledge and consideration of these breed-specific (or even population-specific) differences is important, as this may potentially impact the choice of treatment regimen, prognosis for the patient and advice given to owners of an epileptic dog. In the future breed-specific knowledge and epileptic syndromes may be defined in further detail which may not only advance future research in identifying causative gene mutations but also may support the development of “personalised” or “breed-specific” treatment concepts. The following manuscript reviews dog breeds, which have been identified as being predisposed to idiopathic epilepsy, with a special focus on different epilepsy phenotypes regarding clinical features, treatment response and inheritance. In addition, certain breed-specific diseases that may act as potential differentials for idiopathic epilepsy are highlighted. The seizure terminology used in the original studies has been adapted and uniformed (as far as possible) in line with the new Guidelines for Epilepsy Definition, Classification and Terminology in Companion Animals throughout the manuscript.

**Table 1:** Depicting breed-specific data regarding age of seizure onset

Breed	Age at seizure onset	Reference
Australian Shepherd	2.5 years (median)	Weissl et al. 2012 [9]
Belgian Shepherd	3.3 years (mean)	Berendt et al. 2008 [23]
	3.3 years (mean)	Seppala et al. 2012 [34]
Bernese Mountain dog	26.5 months (mean)	Kathmann et al. 1999 [45]
Border Collie	2.5 years (median)	Hülsmeyer et al. 2010 [8]
Border Terrier	3.2 years (mean)	Kloene et al. 2008 [56]
Dalmatian	2.9 years (median), 3.2 years (mean)	Licht et al. 2002 [65]
English Springer Spaniel	3 years (median)	Patterson et al. 2005 [74]
Finnish Spitz	3 years (median)	Viitmaa et al. 2013 [82]
Golden Retriever	27.5 months (mean)	Srenk et al. 1994 [84]
	24.9 months (mean)	Lengweiler&Jaggy 1999 [86]
Hungarian (Magyar) Vizsla	3 years (median)	Patterson et al. 2003 [87]
Irish Wolfhound	by the age of 3 years in 73 % of dogs	Casal et al. 2006 [24]
Italian Spinone	38 months (mean)	De Risio et al. 2015 [93]
Labrador Retriever	30.6 months (mean)	Jaggy et al. 1998 [95]
	34 months for males and 28 months for females (mean)	Heynold et al. 1997 [94]
	by the age of 4 years in 76 % of dogs	Berendt et al. 2002 [26]
Lagotto Romganolo	6.3 weeks (mean)	Jokinen et al. 2007 [105]
Petit Basset Griffon Vendeen	2 years (median)	Gulløv et al. 2011 [25]
Shetland Sheepdog	predominantly between 1 and 1.5 years	Morita et al. 2002 [109]
Standard Poodle	3.7 years (median)	Licht et al. 2007 [113]
	2.4 years (median), 2.8 years (mean)	Licht et al. 2002 [65]

**Table 2:** Depicting breed-specific data regarding seizure type and seizure remission

Breed	Seizure type	History of cluster seizures or status epilepticus	Remission rate	Reference
Australian Shepherd	36 % generalised epileptic seizures	20 % cluster seizures	12 %	Weissl et al. 2012 [9]
	26 % focal epileptic seizures evolving into generalised seizures	12 % status epilepticus		
	38 % both seizure types	48 % history of both		
	52 % showed also focal epileptic seizures (in addition to their generalised epileptic seizures or focal			
	epileptic seizures evolving into generalised seizures)			
Belgian Shepherd	18 % generalised epileptic seizures	n.s.	n.s.	Berendt et al. 2008 [23]
	25 % focal			
	53 % focal epileptic seizures evolving into generalised seizures			
	4 % unclassified			
	6 % generalised epileptic seizures	n.s.	n.s.	Berendt et al. 2009 [41]
	83 % focal epileptic seizures or focal epileptic seizures evolving into generalised seizures			
	11 % unclassified			
	n.s.	33 % cluster seizures	13.7 %	Gulløv et al. 2012 [37]
	18 % generalised epileptic seizures	33 % cluster seizures	n.s.	Seppala et al. 2012 [34]
	7 % focal epileptic seizures			
	37 % focal epileptic seizures evolving into generalised epileptic seizures			
	34 % generalised epileptic seizures with unknown onset			
	4 % unclassified			
Bernese Mountain dog	98 % generalised epileptic seizures	n.s.	n.s.	Kathmann et al. 1999 [45]
	2 % focal epileptic seizures			
	*Distinction between (primary) generalised epileptic seizures and focal epileptic seizures evolving into generalised seizures was not performed.*			
Border Collie	8 % generalised epileptic seizures	45 % cluster seizures	18 %	Hülsmeyer et al. 2010 [8]
	78 % focal epileptic seizures evolving into generalised seizures	4 % status epilepticus		
	14 % unclassified	49 % history of both		
	45 % showed also focal epileptic seizures (in addition to their generalised epileptic seizures or focal epileptic seizures evolving into generalised seizures)			
Border Terrier	68 % generalised epileptic seizures	n.s.	n.s.	Kloene et al. 2008 [56]
	32 % focal epileptic seizures			
	*Distinction between (primary) generalised epileptic seizures and focal epileptic seizures evolving into generalised seizures was not performed.*			
Cavalier King Charles	39 % generalised epileptic seizures	n.s.	n.s.	Driver et al. 2013 [59]
	36 % focal epileptic seizures			
	25 % focal epileptic seizures evolving into generalised seizures			
Collie (rough and smooth)	Predominantly generalised	35 % cluster seizures	n.s.	Munana et al. 2012 [64]
	*Distinction between (primary) generalised epileptic seizures and focal epileptic seizures evolving into generalised seizures was not performed.*			
Dalmatian	20 % generalised epileptic seizures	63.6 % cluster seizures	n.s.	Licht et al. 2002 [65]
	80 % focal epileptic seizures or focal epileptic seizures evolving into generalised seizures			
English Springer Spaniel	47 % generalised epileptic seizures	38 % cluster seizures	n.s.	Patterson et al. 2005 [74]
	33 % focal epileptic seizures			
	20 % focal epileptic seizures evolving into generalised seizures			
Finnish Spitz	1 % generalised epileptic seizures	16.2 % cluster seizures	n.s.	Viitmaa et al. 2013 [82]
	54 % focal epileptic seizures			
	31 % focal epileptic seizures evolving into generalised seizures			
	7 % generalised with unknown onset			
	7 % completely unclassified			
Golden Retriever	83 % generalised epileptic seizures	n.s.	n.s.	Lengweiler&Jaggy 1999 [86]
	*Distinction between (primary) generalised epileptic seizures and focal epileptic seizures evolving into generalised seizures was not performed.*			
	92 % generalised epileptic seizures	n.s.	n.s.	Srenk et al. 1994 [84]
	*Distinction between (primary) generalised epileptic seizures and focal epileptic seizures evolving into generalised seizures was not performed.*			
Hungarian (Magyar) Vizsla	21 % generalised epileptic seizures	n.s.	n.s.	Patterson et al. 2003 [87]
	62 % focal epileptic seizures			
	17 % focal epileptic seizures evolving into generalised seizures			
Irish Wolfhound	Predominantly generalised epileptic seizures	n.s.	n.s.	Casal et al. 2006 [24]
	*Distinction between (primary) generalised epileptic seizures and focal epileptic seizures evolving into generalised seizures was not performed.*			
Italian Spinone	23 % generalised epileptic seizures		n.s.	DeRisio et al. 2015 [93]
	51 % focal epileptic seizures evolving into generalised seizures			
	26 % generalised epileptic seizures with unknown onset			
Labrador Retriever	24 % generalised epileptic seizures	n.s.	n.s.	Berendt et al. 2002 [26]
	70 % focal epileptic seizures or focal epileptic seizures evolving into generalised seizures			
	91 % generalised epileptic seizures	n.s.	n.s.	Heynold et al. 1997 [94]
	9 % focal epileptic seizures			
	*Distinction between (primary) generalised epileptic seizures and focal epileptic seizures evolving into generalised seizures was not performed.*			
	96 % generalised epileptic seizures	n.s.	n.s.	Jaggy et al. 1998 [95]
	*Distinction between (primary) generalised epileptic seizures and focal epileptic seizures evolving into generalised seizures was not performed.*			
Lagotto Romagnolo	Mainly focal epileptic seizures	n.s.	by 8–13 weeks of age	Jokinen et al. 2007 [105]
Petit Baset Griffon Vendeen	5 % generalised epileptic seizures	n.s.	n.s.	Gulløv et al. 2011 [25]
	41 % focal epileptic seizures			
	52 % focal epileptic seizures evolving into generalised seizures			
	2 % unclassified			
Shetland Sheepdog	Predominantly generalised epileptic seizures	n.s.	n.s.	Morita et al. 2002 [109]
	*Distinction between (primary) generalised epileptic seizures and focal epileptic seizures evolving into generalised seizures was not performed.*			
Standard Poodle	3.5 % generalised epileptic seizures	n.s.	n.s.	Licht et al. 2007 [113]
	33 % focal epileptic seizures			
	60 % focal epileptic seizures evolving into generalised seizures			
	3.5 % generalised epileptic seizures with unknown onset			
	Predominantly focal epileptic seizures or focal epileptic seizures evolving into generalised seizures	34 % cluster seizures	n.s.	Licht et al. 2002 [65]

*n.s.* not specified

**Table 3:** Depicting breed-specific epilepsy prevalence estimates

Breed	Prevalence	Country	Number of dogs	Study information/Epilepsy definition	Reference
Belgian Tervueren	17 %	USA	Complete records of 997 dogs containing 170 epileptic dogs. Data collection late 1980s.	Dogs were included as epilepsy cases when they experienced at least one seizure. Only dogs, which were at least five years of age at the time of the survey were included in the analysis to avoid censoring those individuals, which may have had their first seizure later in life.	Famula et al. 1997 [38]
Belgian Shepherd (Tervueren and Groenendael)	9.5 %	Denmark	1.248 registered dogs in the Danish Kennel Club, representative sample with interview of 516 dog owners identifying 49 epileptic dogs. Data collection 1995–2004.	Dogs that had experienced two or more seizures were defined as epilepsy cases.	Berendt et al. 2008 [23]
Belgian Shepherd (Tervueren and Groenendael)	33 %	Denmark	199 family members (152 Groenendael and 47 Tervueren) containing 66 epileptic dogs (53 Groenendael & 13 Tervueren). Data collection 1988–2005.	Dogs that had experienced two or more seizures were defined as epilepsy cases.	Berendt et al. 2009 [41]
One large family					
Border Terrier	13,1 %	Germany	Records of 365 dogs containing 47 epileptic individuals. Data collection from 1986–2000.	Not provided	Kloene et al. 2008 [56]
Irish Wolfhound	18.3 %	USA	796 Irish Wolfhounds from 115 litters with 146 identified epilepsy cases.	Dogs that had experienced more than 2 seizures. Average inbreeding coefficient (calculated throughout 10 generations) for all the dogs entered into the study was 0.156.	Casal et al. 2006 [24]
Labrador Retriever	3.1 %	Denmark	29.602 Danish Labrador retrievers registered in the Danish Kennel Club in a 10-year period. From the reference population a representative sample of 550 dogs were selected for by random sampling stratified by year of birth. After questionnaire interviews of all 550 dog-owners and clinical investigation of epilepsy suspected dogs, 17 dogs were finally identified with idiopathic epilepsy. Data collection 1989–1999.	Dogs that had experienced two or more recurrent seizures.	Berendt et al. 2002 [26]
Petit Basset Griffon Vendeen (PBGV)	8.9 %	Denmark	876 PBGV dogs registered in the Danish Kennel Club (56 dogs were exported), 471 owner interviews identified 42 epileptic individuals. Data collection 1999–2008.	Dogs that had experienced at least 2 seizures with a minimum interval of 24 h.	Gulløv et al. 2011 [25]
Finnish Spitz Dog	5.4 %	Finland	The epilepsy prevalence was calculated for the dogs that were living when their owners answered a questionnaire (111 epilepsy cases/2.069 total dogs). The questionnaire was sent to all owners of 1- to 10-year-old dogs during the period from June 2003 to July 2004.	Dogs that had experienced at least 2 seizure episodes without interictal neurologic abnormalities.	Viitmaa et al. 2013 [82]
Italian Spinone	5.3 %	UK	The owners of all UK Kennel Club registered Italian Spinoni born between 2000 and 2011 (*n* = 3331) were invited to participate in the study. Of these, 1192 returned the phase I questionnaire and 63 dogs (5.3 %) were identified with idiopathic epilepsy. Of the remaining dogs 0.6 % were identified with structural epilepsy, 0.6 % were identified with reactive epileptic seizures and 1.5 % had unclassified epilepsy.	Recurrent seizures (≥2 seizures occurring >24 h apart) with an onset between 6 months and 6 years of age in dogs with normal interictal physical and neurologic examinations and results of a CBC and biochemistry profile within the normal reference range.	DeRisio et al. 2015 [93]

### Australian Shepherd

In the current literature, there is one specific study of idiopathic epilepsy in Australian Shepherds available [9]. This longitudinal German cohort study was published in 2012 and includes detailed data for 50 affected Australian Shepherds (from Germany, Switzerland and Austria). Fifty unaffected Australian Shepherds served as control dogs. Idiopathic epilepsy was defined as recurrent seizures (≥2 seizures ≥ 4 weeks apart), age at onset ≤ 5 years, unremarkable laboratory results (CBC, biochemical profile, pre- and postprandial serum bile acids concentration), and normal interictal neurologic examination performed by the study investigators or a specialist in veterinary neurology. Magnetic resonance imaging (MRI) and cerebrospinal fluid (CSF) analysis were strongly recommended and obligatory if the age of onset was < 6 months. Dogs with a history of head trauma or structural brain disease were excluded. Advanced diagnostic imaging and CSF analysis were performed in 42 % of affected dogs [9]. Prevalence data for this breed yet are not reported. The median age at seizure onset was 2.5 years [9]. Among epileptic dogs, 64 % were males and 36 % females. The seizure type was specified as (primary) generalised epileptic seizures in 36 % of dogs and as focal epileptic seizures evolving into generalised seizures in 26 % of dogs; the remaining dogs (38 %) showed both seizure types [9]. In addition to their (primary or secondary) generalised seizures, 52 % of dogs also experienced focal seizures [9]. Focal seizures often presented with focal tremors, salivation, dilated pupils, and lateral head turn. Concomitant or solitary episodes with variable states of awareness and behavioural changes like panic attacks, sporadic aggressiveness, pacing, staring, or adverse reactions to emotive words were common [9]. Exclusively discrete seizures (single seizure per day) occurred in 20 % of dogs; of the remaining dogs 20 % had a history of cluster seizures, 12 % had a history of status epilepticus, and 48 % of dogs had a history of both. In summary, 68 % of dogs had a history of cluster seizures and 60 % of dogs a history of status epilepticus [9]. An important observation was that in 28 % of dogs the first seizure event presented as a cluster seizure or status epilepticus. Although severe clinical courses (high incidence of cluster seizures and status epilepticus) were frequently reported among Australian Shepherds, seizure remission was obtained in 12 % of dogs (6 % with and 6 % without antiepileptic treatment) [9], which is in line with remission rates reported in other canine epilepsy studies [8, 10, 11, 27]. The treatment response was reported to be poor (≥1 seizure day/month) in 56 % of Australian Shepherds  (treatment response was only assessed for dogs that were treated sufficiently with phenobarbital alone or in combination with other AEDs; which was the case in 70 % of the study population) [9]. Phenobarbital serum concentrations did not differ between dogs with good seizure control and dogs with poor seizure control [9]. Among the deceased dogs (30 % of the population) the median age at death was 3.1 years [9]. The identification of a causative gene mutation has not yet been reported, but a common founder of 29 affected Australian Shepherds and a clustering manifestation in littermates, full or half siblings was detected [9]. An important observation of this study was, that during the case recruitment phase, a large subset of dogs were reported with recurrent episodes of altered mentation, bizarre behavioural activity and autonomic signs, which corroborated suspicion for potential focal seizures. None of those dogs experienced generalised seizures and in most cases diagnostic work-up was lacking because the neurological signs were mild. Thus, these animals were included as neither case nor control dogs. However, based on those findings and in contrast to the above-mentioned frequent severe clinical courses a distinct mild and focal epilepsy course cannot be excluded for the Australian Shepherd breed and may need to be further elucidated in the future [9]. Identified risk factors: The median age of seizure onset was lower in non-merle-coloured (1.8 years) than in merle-coloured (2.8 years) dogs [9]. Seizure control was associated with age at seizure onset (older age with better seizure control) and coat colour (merle dogs with better seizure control) but appeared unrelated to the ABCB1 (MDR1) genotype (when interpreting the latter finding, it needs to be considered that out of all the epilepsy cases  only one dog was determined to be homozygous for the ABCB1-1Δ mutation)  [9]. Seizure remission occurred independently of the clinical course and seizure frequency [9]. No association was found between seizure control, phenobarbital serum concentration and the number of administered drugs, indicating that there may be a subcategory of severe intrinsic epilepsy in Australian Shepherd dogs [9]. Reduced survival times were found in dogs with poor seizure control, in dogs <2 years of age at seizure onset, in dogs experiencing ≥ 10 seizure days within the first six months after seizure onset and in non-merle coloured dogs [9]. However, in a multivariable COX regression analysis only a high initial seizure frequency (≥10 seizure days/6 months after seizure onset) and poor seizure control remained statistically significant with respect to reduced survival times. Overall, dogs with good seizure control had a lower risk of death than dogs with poor seizure control [9]. Potential breed-specific diseases that may mimic idiopathic epilepsy: Neuronal ceroid lipofuscinosis (NCL), a neurodegenerative storage disorder, may also manifest with epileptic seizures and/or fly-biting episodes and therefore may present a potential differential in young Australian Shepherds with seizures. However, NCL usually manifests with concurrent severe neurological and/or cognitive abnormalities and seizures usually occur late in the disease course [28]. A gene test for NCL in Australian Shepherds is available (missense mutation in the CLN6 gene) [29]. One study from 2011, diagnosed two Australian Shepherds with exercise-induced collapse (EIC), which also may mimic a seizure event, and therefore should be considered as a potential differential to epileptic seizures. However, a dynamin-1 (DNM1)-gene-mutation was not detected in those two Australian Shepherds [30]. EIC usually is triggered by strenuous physical exercise and consciousness usually remains preserved during episodes, which may help to clinically differentiate between epileptic seizures and EIC [30]. Furthermore, the Australian Shepherd breed has a high frequency of the ABCB1-gene mutation (nt230 (del4)) that results in non-functional P-glycoprotein (P-gp) expression and neurotoxicity to drugs, which are P-gp substrates [31, 32]. This may need to be considered as a differential for idiopathic epilepsy  in Australian Shepherd dogs that present with acute epileptic seizures and potential previous exposure to neurotoxic P-gp substrates. The frequency of dogs homozygous for the mutant allele is reported to range between 1.7 − 25 % depending on the respective study and geographic area [33].

### Belgian Shepherd (mainly Groenendal and Tervueren variants)

There are ten different studies available that focus specifically on idiopathic epilepsy in the Belgian Shepherd (mainly Groenendal and Tervueren variants) [23, 34–42]. This relatively high number of studies leaves the Belgian Shepherd as one of the most intensively studied dog breeds in the field of canine epilepsy. Interestingly, an inheritance of idiopathic epilepsy in this breed was first suggested in 1968 [42]. All of the available studies respectively focus on seizure semiology, prevalence, mode of inheritance and gene mutation identification; and have been conducted mainly in Denmark [23, 37, 41], the United States [35, 36, 38–40] and Finland [34]. The variability in results between individual studies may be attributed to the examination of geographically and genetically distinct populations and variable study designs and inclusion and exclusion criteria being applied. One Danish study published in 2008 was an epidemiological study of a larger population of Belgian Shepherds registered in the Danish Kennel Club in a 10-year period. Prevalence was estimated at 9.5 % based on interviews of 516 dog owners, which identified 49 dogs with idiopathic epilepsy [23]. Mean age at seizure onset was 3.3 years. However, 39 % of all affected dogs did not experience their first seizure until after four years of age. Of the investigated epileptic dogs, 63.3 % were females and 36.7 % were male; however, a significant gender predisposition was not detected. The seizure type was reported to be focal in 25 % of dogs, focal evolving into generalisation in 53 % of dogs and (primary) generalised in 18 % of dogs (in 4 % of dogs seizures could not be classified). The most commonly reported focal seizure phenomenology included ataxia, crawling, swaying, behaviour suggesting fear, salivation, excessive attention seeking and disorientation. The median survival time from seizure onset was 2.5 years among deceased dogs [23]. In 2009 the same authors investigated a selected large Danish Belgian Shepherd family including 199 individuals with 66 idiopathic epilepsy affected dogs [41]. The epilepsy prevalence in this selected family was estimated at 33 % [41], which showed that accumulation of epileptic individuals within certain breeding lines can result in substantially higher prevalence estimates than in the breed in general (as reflected by the two Danish studies) [23]. As found in the Danish breed study from 2008, the seizure type was predominantly (83 % of dogs) defined as focal or focal epileptic seizures evolving into generalised seizures, while only 6 % of dogs experienced (primary) generalised epileptic seizures [41]. In 11 % of dogs, seizures could not be classified. Due to the high prevalence of focal seizures the authors discussed if this familial epilepsy could be compared to familial focal epilepsy in humans [41]. This large Danish Belgian shepherd family was further investigated with respect to survival and selected risk factors for premature death by a longitudinal observational study published in 2012 [37]. The life span of epileptic dogs was not significantly shortened by the presence of epilepsy. Epilepsy was the predominant cause of death in the population and epilepsy-related deaths accounted for 70 % of all deaths in the group of dogs with epilepsy. Two probable sudden unexpected deaths related to epilepsy occurred in dogs with generalised seizures. Cluster seizures occurred in 33 % but did not significantly influence the life span of epileptic dogs. Dogs with epilepsy had an epilepsy remission proportion of 13.7 % [37]. A 2012 genome wide-association study including Belgian Shepherd epilepsy cases from Denmark, Finland and USA (159 cases and 148 controls) identified a novel idiopathic epilepsy locus [34]. The mean age at seizure onset in the dogs participating in the study was 3.3 years [34], which is in line with findings of the Danish study [23]. The median seizure frequency was 5.25 seizures per year with some dogs experiencing less than one seizure per year and others up to 10 seizures per day. One third (33 %) of the affected dogs had a history of cluster seizures. The seizure type was defined as focal epileptic seizures evolving into generalised seizures in 37 % of dogs, as generalised seizures with unknown onset in 34 % of dogs, as (primary) generalised in 18 % of dogs and as focal in 7 % of the dogs. In 4 % of dogs the seizures remained unclassified [34]. Only 3 % of the dogs did not respond to antiepileptic drug treatment, while the remaining dogs all responded at least with some degree of seizure frequency reduction. A number of dogs participating in the study had an EEG examination and interictal EEG revealed epileptiform activity with variable foci in all examined dogs [34]. Multiple studies have been conducted with respect to potential modes of inheritance in this breed, however results were not always consistent and this again may reflect different study design, inclusion and exclusion criteria. In the Danish study from 2009 the mode of inheritance of epilepsy was based on segregation analysis reported to be simple mendelian with a segregation pattern resembling autosomal dominant inheritance but with possible incomplete penetrance [41]. These results contradict the findings from an older USA study in 2003, in which a polygenic mode of inheritance influenced by a single autosomal recessive locus was suggested [35]. Furthermore a study from 1997 found that a single locus model does not appear adequate as an explanation [38], but the same investigators in a 2000 study suggested a single locus with a large effect on the incidence of seizures [39]. The 1997 USA study estimated the heritability of epilepsy in the Belgian Shepherd dog at 0.77 [38], and the 1998 USA study predicted that the offspring of the mating of two non-epileptic dogs has a probability of 0.99 of never suffering from a seizure, while the offspring of the mating of two dogs who have each had one seizure has a predicted probability 0.58 of never suffering from a seizure [40]. Although, the clinical phenotype of idiopathic epilepsy in the Belgian shepherd is well described and extensive research efforts have been undertaken, it has not yet been possible to identify causative gene mutation(s) responsible for idiopathic epilepsy in the breed [34–36]. Identified risk factors: Intact dogs with idiopathic epilepsy had a significantly increased risk of being euthanised because of idiopathic epilepsy compared to neutered dogs with idiopathic epilepsy [23]. Recently, it was found that homozygosity for a two-SNP haplotype within the ADAM23 gene conferred the highest risk for idiopathic epilepsy among the investigated Belgian shepherds [34]. These data suggested that the identified ADAM23 variant is a polymorphism, but yet needs to be further confirmed. Potential breed-specific diseases that may mimic idiopathic epilepsy: In the authors’ experience the most important differential to be excluded in the Groenendael and Tervueren is a (often exercise induced) episodic involuntary movement disorder similar to paroxysmal dyskinesia described in Chinook dogs [43] and Border terriers [44]. It is recommended that the paroxysmal episode be filmed. The paroxysmal movement disorder can be distinguished from seizures because the dogs remain responsive to stimuli and their environment, for example will continue to try to play. The episodes are typically longer in duration than epileptic seizures and characterised by dystonic limb lifting (all joints in flexion). The dog may become recumbent but often remains standing (personal communication Clare Rusbridge February 2015).

### Bernese Mountain dog

In the current literature, one study about idiopathic epilepsy in Bernese Mountain dogs is available [45]. This study includes 50 affected dogs from Switzerland and was published in 1999. Idiopathic epilepsy was diagnosed when physical and neurological examination, haematology, serum biochemistry, urine and CSF analysis were unremarkable. Detailed data regarding definition of idiopathic epilepsy were not provided in this study [45]. Sixty-nine healthy Bernese Mountain dogs served as control dogs and a possible gender predisposition was analysed by the use of a non-preselected population containing 4005 Swiss Bernese Mountain dogs [45]. Prevalence data have not been reported for this breed [45]. The mean age of seizure onset was 2.2 years (26.5 months) with 62 % of dogs exhibited their first seizure between one to three years of age, 20 % had an age of seizure onset of less than one year and 18 % experienced the first seizure at an age ≥ 3 years [45]. A gender predisposition for males (62 %) compared to females (38 %) was observed. The gender ratios (males to females) were 1.6:1 among epileptic dogs, 1:1.1 among non-epileptic control dogs and 1:1.4 among all dogs [45]. The seizure type was defined as generalised in 98 % of dogs and as focal in 2 % of dogs [45]. However, a detailed differentiation between (primary) generalised seizures and focal epileptic seizures evolving into generalised epileptic seizures was not conducted, hence some of the dogs may have experienced focal seizures evolving into generalisation rather than (primary) generalised seizures. The seizure frequency was not analysed in detail, but was reported to range from three seizures per week to one seizure every year, with 50 % of dogs experiencing more than one seizure every two months. The results of the pedigree analyses and binomial test were best compatible with a polygenic, recessive (possibly sex-modified) mode of inheritance [45]. The identification of a causative gene mutation has not yet been reported [45]. Identified risk factors: The age at seizure onset was significantly lower in dogs from affected parental animals than in dogs from healthy parental animals [45].

### Border Collie

In the current literature, one specific study about idiopathic epilepsy in Border Collies is available [8]. This study – conducted in Germany – was published in 2010 and provides data regarding clinical characteristics and heritability of epilepsy among a German Border Collie population [8]. The latter study, included data of 49 Border Collies diagnosed with idiopathic epilepsy; no control dogs were included. Idiopathic epilepsy was defined as recurrent seizures (≥2 seizure days at least 4 weeks apart) with an onset between 6 months and 5 years of age in dogs with otherwise normal physical, laboratory, and neurological characteristics upon examination. Requested minimal laboratory investigations included a CBC and biochemical profile. MRI and CSF analysis were requested if age at seizure onset was < 6 months or > 5 years of age [8]. Detailed prevalence data have yet not been provided for this breed [8]. Although detailed prevalence data are not available, the Border Collie was among the most common affected breeds in several epidemiological canine epilepsy studies in the UK [17, 46, 47]. The median age at seizure onset in the German study was 2.4 years with 74 % of dogs experiencing their first seizure between 1 and 5 years. However 18 % experienced the first seizure at an age ≤ 1 year and 8 % of dogs had an age of ≥ 5 years. No gender predisposition was detected with 49 % males and 51 % females. The seizure type was defined as focal epileptic seizures evolving into generalised seizures in 78 % of dogs and as (primary) generalised in 8 % of dogs. In 14 % of dogs the seizures remained unclassified, as seizure onset was not clearly observed. In addition, 45 % of dogs also had sporadic focal epileptic seizures, which manifested as sudden uncontrolled head or facial twitching mostly associated with impaired consciousness. Active epilepsy (≥1 epileptic seizure in the last year of the study or in the year preceding death) was documented in 82 % of dogs and seizure remission was reported for 18 % of the Border Collies with idiopathic epilepsy, which is similar to reported remission rates in other dog populations [9, 11, 27, 47]. However, a recent canine epilepsy study which focussed on identification of clinical risk factors for remission revealed the Border Collie as the breed least likely to achieve seizure remission [47]. Of all affected dogs 45 % had a history of cluster seizures, 4 % had a history of status epilepticus and 49 % had a history of both. Overall, 94 % of all dogs included in the German study experienced at least one episode of cluster seizures and 60 % of all dogs had at least one episode of status epilepticus [8]. A high prevalence of cluster seizures among Border Collies was also found in a recent study about canine juvenile epilepsy in the UK [10], however, in contradiction to this data another UK study reported the Border Collie as being less affected by cluster seizures (>80 % not clustering) [46]. The median age at death among the deceased epileptic Border Collies (47 % of the study population) was 5.2 years [8], which is more than half that of the general UK Border Collie population (median age at death 13.5 years) found in another study [48]. The median survival time from seizure onset for deceased epileptic Border Collies was short being only 2.1 years in the German study [8]. This finding was supported by another study that also found a significant shorter mean survival time for Border Collies with idiopathic epilepsy (3.6 years) compared to a general dog population with idiopathic epilepsy (7.9 years) [10]. Treatment response was reported to be poor (≥1 seizure day/month) in 71 % of dogs that were treated adequately (67 % of the study population) with ≥ 2 antiepileptic drugs [8]. In summary, all above-mentioned clinical data suggest that this breed may generally have a severe epilepsy course and epileptic Border Collies are more likely to be euthanised than affected dogs of other breeds. Based on pedigree analysis, 29 affected dogs shared a common ancestor, indicating a strong genetic background for epilepsy in Border Collies. The identification of a causative gene mutation has yet to be achieved [8]. Identified risk factors: No positive impact of neutering on the course of epilepsy was detected. Comparison between dogs with active epilepsy and dogs in remission identified significant differences in age at seizure onset (older age at seizure onset in dogs that went into remission) and age at death (younger age at death for dogs with active epilepsy). Furthermore, initial seizure frequency (during the first 6 months) was significantly lower in dogs that went into remission compared to dogs with continuing epilepsy. Reduced survival times were found in dogs with young age at seizure onset (≤2 years of age) and dogs with a severe epilepsy course (occurrence of status epilepticus) [8]. No significant association between survival time and sex, reproductive status or number of administered drugs was identified. A Swiss study identified a polymorphism in the ABCB1-gene that was found to be associated with antiepileptic drug resistance (T > G variation in intron 1) in Border Collies [49]. This ABCB1-polymorphism (T > G variant) was detected in a later Japanese study with a frequency of 9.8 % among a Japanese Border Collie population [50]. In contrast to a known “loss of function” for the nt230 (del4) mutation, the T > G variation is hypothesised to have an ABCB1 drug transporter”gain of function” and therefore potentially might contribute to drug resistance. However, future research is required to investigate those associations further and also to investigate the possible mechanisms of this ABCB1-polymorphism on drug resistant epilepsy in Border Collies. Potential breed-specific diseases that may mimic idiopathic epilepsy: NCL, a neurodegenerative disorder, may also manifest with epileptic seizures and therefore may present a potential differential in young Border Collies presenting with seizures [51–53]. However, NCL is reported to manifest between the age of 15 and 22 months [51, 53] with severe and rapidly progressive neurological signs (e.g. vision impairment, gait abnormalities, dementia, behavioural abnormalities, aggression). Affected Border Collis died before the age of 3 years and the latest they were euthanized after the onset of clinical signs was 6 months [51]. A causative gene mutation for NCL in Border Collies has been identified and a gene test is available (mutation in the CLN5 gene) [54]. One study from 2011 reported 20 Border Collies diagnosed with exercise-induced collapse, but a DNM1-gene-mutation was not observed in any of these Border Collies [30]. However, in contrast to epileptic seizures, EIC usually is triggered by strenuous exercise and consciousness usually remains preserved during episodes and the dog’s behaviour suggests discomfort from heat (panting, seeking shade and/or water), which may help a clinician to differentiate between these two diseases [30]. The Border Collie breed is also affected by the ABCB1/MDR1-gene mutation (nt230 (del4)), which may need to be considered in dogs with acute seizures and potential previous exposure to neurotoxic P-gp substrates. The frequency of homozygous affected dogs is reported with 0.3 % [33]. There is one case report describing hyperammonemic encephalopathy (normal bile acids, but abnormal ammonia tolerance test) secondary to a hereditary selective cobalamin malabsorption in a juvenile Border Collie that presented with neurological signs, such as an abnormal mental state (stupor) [55]. However, although this particular dog did not seizure, hyperammonemia may carry a potential risk for reactive seizures in other Border Collies with such hyperammonemic encephalopathy.

### Border Terrier

One specific study about epilepsy in Border Terriers that was conducted in Germany and published in 2008 is available in the current literature [56]. This study included 47 affected dogs and 318 non-affected control dogs collected by questionnaires send to owners of dogs registered in the German Terrier Club. Detailed data regarding epilepsy definition were not provided [56]. The prevalence of epilepsy was estimated at 13.1 % among the investigated population [56]. In line with this high prevalence, a UK epilepsy prevalence and risk factor study reported the Border Terrier with a 2.7 times increased odds of epilepsy compared with crossbred dogs [17]. A gender predisposition was not detected with 53 % males and 47 % females in the German study. The mean age at seizure onset was 3.2 years. The clinical course was assessed as mild in most of the dogs (70 %) with only occasional seizures per year; only 27 % of dogs suffered from multiple seizures per month. Cluster seizures were documented in 8.5 % of dogs. The seizure type was defined as generalised epileptic seizures in 68 % of dogs and as focal epileptic seizures in 32 % of dogs, however a differentiation between (primary) generalised epileptic seizures and focal epileptic seizures evolving into generalised seizures was not performed. In 17 % of dogs autonomic signs (urination, defecation) were reported during a seizure and some of the dogs (27.6 %) experienced preictal signs, such as restlessness, disorientation, behaviour suggesting fear or seeking owners’ attention [57]. Hence, according to the new classification guidelines some of those dogs with a “preictal phase” (27.6 %) might be reclassified as having focal seizures evolving into generalised seizures. The seizures were defined as tonic in 46.8 % of dogs, as clonic in 14.9 % of dogs and as tonic-clonic in 38.3 % of dogs. In 51 % of dogs seizures occurred when they were in a resting position [57]. One important finding was that in most of the dogs (79 %) consciousness was assessed as preserved during a seizure, and only 21 % of dogs experienced an ictal loss of consciousness. Thirty per cent of dogs were treated with phenobarbital, and in 23.4 % of dogs, owners subjectively reported at least some clinical improvement [57]. The identification of a causative gene mutation has not yet been reported [56]. Potential breed-specific diseases that may mimic idiopathic epilepsy: Canine epileptoid cramping syndrome (CECS) is proposed as paroxysmal dyskinesia affecting Border Terriers. Recently, detailed data regarding typical clinical phenotypes of 29 CECS-affected Border Terriers have been published [44]. Based on the similarities regarding clinical semiology and typical age of disease manifestation, CECS is an important differential to epileptic seizures in this breed; even for experienced clinicians CECS may mimic a seizure disorder and vice versa. Distinct phenotypic characteristic of the paroxysmal events in CECS are generalised tremor, dystonia and difficulty walking. Episodes were reported to last from 2–30 min in the majority of dogs and in some up to 150 min [44]. In addition, some owners report signs of gastrointestinal distress associated with the episodes, including borborygmi during the episode and vomiting and diarrhoea before or after the episode in almost 50 % of the dogs. From a clinical point of view, the assessment of consciousness and the occurrence of autonomic signs such as urination, defecation or salivation during an episode may help to differentiate between both diseases, with CECS usually being characterized by a normal conscious state and absence of above mentioned autonomous signs [44]. However, as 79 % of epileptic Border Terriers were reported to have preserved consciousness during an epileptic seizure in the German study [56] and 62 % of Border Terriers diagnosed with CECS showed some kind of “pre- and postcramping” signs (such as eating grass, vomiting, or seeking to be near owners) [44] diagnosis still may remain challenging and potential overlapping of both diseases needs to be further elucidated in the future (e.g. with ambulatory ictal EEG examinations). With regard to response to therapy, therapeutic trials with phenobarbital, potassium bromide, diazepam, butylscopolamin resulted in no improvement in the majority of CECS affected dogs, but 50 % of the owners felt improvement after a dietary change (e.g. change to hypoallergenic diet) [44].

### Cavalier King Charles Spaniel

For several years it has been suggested that idiopathic epilepsy may occur as an independent disease in this breed and may not be the consequence of the frequently occurring Chiari-like malformation [58]. This hypothesis was supported by a study from 2013 that did not find a significant association between the degree of the Chiari-like malformation, (such as degree of ventriculomegaly) and the occurrence of epileptic seizures [59]. However, an overlapping of the two diseases cannot be entirely excluded. According to the findings of the study published in 2013 the seizure type was defined as (primary) generalised epileptic seizures in 39 % of the dogs, as focal epileptic seizures in 36 % and as focal epileptic seizures evolving into generalised seizures in 25 % of dogs [59]. No detailed data regarding potential modes of inheritance are available; however, epilepsy was found more frequently in lines originating from whole-colour dogs [58]. Potential breed-specific diseases that may mimic idiopathic epilepsy: Cavalier King Charles Spaniels are also known to suffer from Episodic Falling (paroxysmal exercise-induced dyskinesia) [60]. Episodic falling is a movement disorder that typically manifests between the age of 4 months and four years. Falling episodes are induced by physical activity, stress and excitement and manifest with hypertonicity of the limbs resulting in inability to move or even complete collapse. In contrast to epileptic seizures consciousness usually is not affected during these episodes [60]. A gene test is available for episodic falling that is based on evidence of a BCAN (brevican) mutation [61, 62]. Older Cavalier King Charles spaniels (>5 years old) have a high prevalence of myoclonus, which manifests most commonly as a brief jerking of the head and forelimbs when the dog is standing or sitting. Initially the syndrome is relatively benign but can be progressive with affected dogs suffering frequent jerks which may interfere with function, for example cause the dog to fall or stumble [63]. The syndrome can be confused with focal epileptic seizures but generally does not respond to AEDs licenced for dogs although may respond to levetiracetam (personal communication Clare Rusbridge February 2015). The pathogenesis of the myoclonus is as yet undetermined.

### Collie (rough and smooth coated; or Scottish sheepdog also known as Scottish Collie)

There is no specific epidemiological study for the Rough and Smooth coated Collie available, but one study has been published, which specifically focused on seizure control in association to the ABCB1/MDR1-genotype in epileptic Rough and Smooth coated Collies [64]. This study was conducted in the USA and included 29 Rough and Smooth Collies with suspected idiopathic epilepsy [64]. The investigated population consisted of 25 Rough coated Collies, 3 Smooth coated Collies, and 1 Collie cross. Collies with an age of seizure onset between 6 months and 5 years of age and a minimum 6-month history of AED administration were recruited for this cohort study [64]. All dogs had a presumptive diagnosis of idiopathic epilepsy, which was made by the primary veterinarian based on examination findings and laboratory analysis [64]. A good seizure control was defined as ≤ 1 seizure/month and no occurrence of cluster seizures; a poor seizure control was defined by > 1 seizure/month or the occurrence of cluster seizures [64]. Among the investigated population 66 % received one antiepileptic drug, 31 % received two antiepileptic drugs and 3 % received three antiepileptic drugs. Overall, 38 % of dogs were reported to have a poor seizure control, with a mean seizure frequency of 3.9 seizures/month; the remaining 62 % of dogs had a good seizure control with a mean seizure frequency of 0.29 seizures/month. Eighty-nine per cent of dogs with a good seizure control were managed with a single AED and 50 % of those dogs with a good seizure control became seizure free. Of the dogs with a poor seizure control 91 % had a history of cluster seizures (=35 % of all dogs). Of all participating dogs, 48 % were homozygous for the ABCB1-/MDR1-gene mutation (nt230 (del4)) (M/M), 38 % were heterozygous for the mutation (M/N), and 14 % had the wild-type genotype (N/N). The M/M group had significantly better seizure control than the M/N or N/N groups. However, as the M/N and N/N groups suffered more frequently from antiepileptic drug adverse effects than dogs from the M/M group (with non-significant differences of antiepileptic drug serum concentrations among all groups), the authors concluded, that the association of the M/M genotype with better seizure control compared to the M/N or N/N (with poorer seizure control) does not support the transporter hypothesis of Pgp-mediated drug resistance in this breed. The authors rather considered the association between genotype and seizure outcome as an epiphenomenon, with the ABCB1/MDR1-gene mutation being associated with a less robust seizure phenotype that favours drug efficacy in accordance to the intrinsic disease severity theory [64]. However, Rough and Smooth coated Collies appear to have a less severe clinical manifestation of idiopathic epilepsy when compared with other breeds such as the Australian Shepherd or Border Collie. Potential breed-specific diseases that may mimic idiopathic epilepsy: As mentioned above Rough and Smooth Collies are breeds affected by ABCB1/MDR1-gene mutation, which needs to be considered for dogs with very acute seizures and potential previous exposure to neurotoxic P-gp substrates. The frequency of homozygous affected dogs is reported to range between 24 − 52 % depending on the respective study and geographic area [33].

### Dalmatian

To date, there is no study available that has specifically evaluated idiopathic epilepsy in Dalmatians, but Short et al. found that the Dalmatian was in the “top 14” of dog breeds with epilepsy in the UK [46]. By contrast the Dalmatian was not listed in the “top 20” of dog breeds ranking in Kennel Club number registrations in 2011, which suggests a predisposition to epilepsy [46]. Furthermore, one study that reported clinical manifestations of naturally occurring canine epileptic seizures also included very detailed seizure data of 11 Dalmatians with probable idiopathic epilepsy [65], and hence this breed was included in the present manuscript. However, interpretation of these data should be made with caution due to the low number of investigated dogs. The latter study classified dogs as having probable idiopathic epilepsy if they had had at least 1 seizure without any evidence of an underlying cause. Specifically, the following conditions all had to be met: the owner’s answers to health- related questions revealed no illnesses or events (e.g., head trauma) that could plausibly account for the seizures, at least 1 year had passed since seizure onset during which no interictal neurologic abnormalities were observed, and the dog was between 6 months and 7.5 years old when seizures began [65]. The median age at seizure onset among those eleven Dalmatians was 2.9 years. The number of Dalmatians was too small to reliably assess a potential gender predisposition, however, 36.4 % of the dogs were males and 63.6 % were females [65]. Among dogs, in which owners were able to reliably report the initial stages of an epileptic seizure, 20 % were reported to have (primary) generalised seizures and 80 % were reported to suffer from focal or focal seizures evolving into generalisation [65]. The mean seizure frequency was analysed with 9.7 seizures per year. At least one cluster seizure event was reported in 63.6 % of dogs; further analysis revealed that among dogs with cluster seizures the mean percentage of total episodes that were clusters was 17.8 % [65]. Mean duration of a generalised seizure was 3.3 min, whereas mean duration of a focal seizure was 4.7 min. Mean duration of a postictal phase after generalised seizures was 16 min and after a focal seizure 0.9 min [65]. The majority of dogs (72.7 %) received antiepileptic treatment, but treatment response could not be assessed reliably because few dogs provided enough seizure baseline data, making it impossible to evaluate their overall response to treatment [65].

### Dutch breeds

There are nine different Dutch pedigree breeds. Although most of these breeds occur in the surrounding countries of the Netherlands, all nine breeds are small in number, increasing the risk of hereditary disorders [66, 67]. Recently all case-record logs from the nine Dutch breeders associations representing the nine Dutch breeds were reviewed [68]. Dogs presented with epileptic seizures were either classified as suffering from idiopathic generalised tonic-clonic seizures or classified as focal epilepsy based on their history, the clinical signs and diagnostic work-up. There are four breeds, ‘Het Nederlandse Kooikerhondje’ ‘Drentse Patrijshond’, ‘Stabyhoun’, and ‘Saarlooswolfhond’ with a higher incidence of idiopathic epilepsy [68]. Interestingly the incidence of idiopathic epilepsy is, within the other Dutch breeds, ‘Hollandse Herder (also called Dutch Shepherd)’, ‘Smoushond’, ‘Het Nederlandse Markiesje, ‘Wetterhoun’, ‘Nederlandse Schapendoes’ remarkably low compared with most pedigree breeds. Hollandse herder: the incidence of generalised tonic-clonic epilepsy varied during the last ten years around 0.25 %. The affected dogs are presented between one to three years of age [68]. This breed, that originates to before 1890, may have a small common ancestry with the Belgian Shepherd dog [66] and like the Belgian Shepherd dog different hair coat varieties are recognised (shorthair, longhair and roughcoat). However, in contrast with the Belgian Shepherd the incidence of idiopathic epilepsy is low in all varieties. Smoushond: the incidence of dogs presented with generalised tonic-clonic seizures was 0.7 % during the last 20 years. Another 0.7 % of the dogs were presented with signs suggestive of focal epilepsy but none of these dogs showed secondary generalisation As the frequency of these focal seizures remained low in these dogs it remains questionable how to classify these dogs [68]. Het Nederlandse Markiesje: the incidence of idiopathic epilepsy appears to be, with only 0.29 %. Within this breed a novel lethal neurological disorder has been identified in recently weaned pups, with an incidence of 1 %, that has been classified as a paroxysmal hyperekplexia [69]. Although affected dogs remain conscious, the tonic rigidity of this disorder may be confused with a myoclonic or tonic epilepsy [69]. Wetterhoun: this extremely small breed belongs to the group of Retrievers and Water dogs and originated from the Friesian area of the Netherlands. Although the number of Wetterhouns bred annually is too small (between 60 an 150) to maintain a healthy population, the incidence of generalised tonic-clonic epilepsy is extremely low: 0.1 %. Similar figures have been found in the Nederlandse Schapendoes (0.18 %) [68]. In contrast with these aforementioned five breeds that have a very low prevalence of epilepsy, there are four other Dutch breeds that are more greatly affected. The highest incidence has been found in the Drentse Patrijshond. Within the Drentse Patrijshond, a spaniel type of hunting dog, idiopathic epilepsy has been reported since 1986 [70]. In 1986 the incidence of idiopathic generalised epilepsy was at least 1.4 %. If the investigators excluded a group with missing data the incidence was calculated to be up to 9.4 %. Males and females were equally affected, and the dogs, were between 9 months and 5 years of age with a median of three years of age at onset of seizures [70]. Affected dogs did not have a higher inbreeding coefficient compared to the non-affected population [70]. The hereditary grade (h^2^) was found to be between 0.33 and 0.47, which is highly suggestive for a genetic origin. Recently, the number of affected animals was again evaluated using the earlier described inclusion criteria [68]. The incidence, calculated over the last 20 years currently varies between 3 to 5 %. The majority of the affected dogs only have one to two seizures per time period but a small number (<10 %) suffered from clusters with more than three seizures per event [68]. In contrast with the earlier study of Bobbert and Reekers [70] , dogs could be presented up to 8 years of age before being identified as suffering from idiopathic epilepsy. As some of these affected dogs already had been bred from, the incidence of idiopathic epilepsy remains high. The Stabijhoun, a Friesian type of spaniel hunting dog (also called ‘moles dog’ as it is used to catch moles) had an average incidence of 1.5 % over the last 15 years. Dogs were presented between one and 5 years of age. Although not statistically significant males appeared to be more affected than females (59 % males to 41 % females). Typically, the dogs are presented with generalised tonic-clonic seizures. Het Nederlandse Kooikerhondje, also called ‘Dutch Decoy Dog’ as it is used as “decoy” to catch ducks [71] was re-established after the 2nd World War and subjected to a long period of intense inbreeding [72]. As a consequence several, hereditary, neurological disorders have been recognized in this breed [73]. The incidence of idiopathic epilepsy, calculated for the last 14 years is estimated to be 1.4 %. Males (71 %) appear to be overrepresented compared to females (29 %). The dogs are normally presented between the age of 1 and 3 years old [68]. The last breed, the Saarlooswolfhond, is a breed established from a German Shepherd and European wolf hybrid and was created just before the 2^nd^ World War by Mr. Leendert Saarloos [66, 67]. The population is very small and highly inbred, with inbreeding coefficients varying between 25 to 60 %. A total of 37 dogs have been identified suffering from tonic-clonic seizures. The treatment response rate appears to be poor in this breed and up to 50 % suffer from cluster seizures. Up to 50 % of these dogs were euthanised, due too poor control of the seizures, within two years after their first seizure [68]. As the breed is highly inbred, selection against epilepsy is very challenging. Currently the breeders are using, with permission of the Dutch Kennel club, outcrossing to improve the genetic variation within this breed [68].

### English Springer Spaniel

In the current literature, one specific study about epilepsy in English Springer Spaniels is available [74]. This study was published in 2005 and provides data regarding clinical characteristics and mode of inheritance of idiopathic epilepsy among an US English Springer Spaniel population [74]. The latter study included 45 dogs diagnosed with idiopathic epilepsy. Idiopathic epilepsy was defined as ≥2 seizures at least 1 month apart, without any evidence of toxin exposure or head trauma, and results of routine serum biochemical testing and interictal neurologic examinations were normal. Dogs in which seizures first began at < 6 months or > 5 years of age were considered to have idiopathic epilepsy only if CSF analysis and CT or MRI had been performed and no underlying cause of the seizures had been identified or if 2 years had lapsed since the onset of seizures without any interictal neurologic abnormalities [74]. The median age of seizure onset was reported as 3 years. A bimodal age distribution was detected with one peak at 1–3 years (60 %) and one peak at 5–6 years (20 %) [74]. There was no significant gender predisposition [74]. The seizure type was defined as (primary) generalised in 47 % of dogs and as focal or focal epileptic seizures evolving into generalised seizures in 53 % of dogs. For the dogs with focal seizures, 58 % of dogs had simple focal seizures, 38 % had focal epileptic seizures evolving into generalised seizures, and 4 % had complex focal seizures characterized by stereotypic repetitive behaviours [74]. Applying the new seizure classification guidelines the seizure type distribution would be: 47 % generalised epileptic seizures, 33 % focal epileptic seizures and 20 % focal epileptic seizures evolving into generalised epileptic seizures. Seizure frequency ranged from 12 seizures per month to 1 seizure every 2 years (median 5 seizures per year). A history of cluster seizures was reported in 38 % of dogs [74]. Sixty-seven per cent of the dogs received an antiepileptic drug treatment. Treatment response was assessed subjectively based on the owners‘opinion with a reported good response in 23 % of treated dogs, a moderate response in 47 % of dogs and a poor response in 30 % of dogs [74]. Detailed prevalence data have yet not been provided for this breed. However, one epidemiological study conducted in the UK reported 2.3 % English Springer Spaniels among 1260 dogs with epilepsy and reported a high incidence of cluster seizures [46]. Similarly, another UK study found that the English Springer Spaniel was one of the most commonly epilepsy affected purebreds [17]; however, it must be considered that the English Springer Spaniel is a popular breed in the UK, and thus may be overrepresented in this population in general. In contrast to the UK epidemiological studies, the English Springer Spaniel 2013 UK Breed Health survey  found that the prevalence of epilepsy was 0.6 % (26 of 4327 dogs) [75]. Epilepsy was reported as occurring in young and middle aged dogs, of which 18 were male and 8 female. However in the mortality section of this survey (dogs which had died between 1st January 2008 and 31st July 2013) epilepsy was reported as the cause of 3.2 % of all deaths [75]. Many of the deaths were young dogs and consequently the UK breed club expressed concern about the disease and its impact. Pedigree analysis and results of segregation analysis of the US study were consistent with a partially penetrant autosomal recessive or polygenic inheritance [74]. The identification of a causative gene mutation has not yet reported [76]. Potential breed-specific diseases that may mimic idiopathic epilepsy: Fucosidosis is a lysosomal storage disease, which affects humans and English Springer Spaniels. The disease is autosomal recessively inherited in both species and results from a deficiency of the enzyme alpha-L-fucosidase [77, 78]. Affected English Springer Spaniels present with behavioural changes and signs of motor dysfunction that start at one to two years of age. Behavioural changes may manifest as bizarre behaviour patterns, aggression or unusually depressed mental state, and affected dogs appear to forget previously learned behaviours [77]. These behavioural changes may carry a risk of mistaking focal epileptic seizures as a potential differential in the early stage of the disease. However, Fucosidosis progresses rapidly, and death or euthanasia usually occurs within a few weeks from the onset of clinical signs [78]. A genetic test for Fucosidosis is available [79].

### Finnish Spitz

Four specific studies about epilepsy in Finnish Spitz dogs are currently available [80–83], reporting prevalence, clinical characteristics, mode of inheritance, imaging findings and EEG findings. One prospective epidemiological study published in 2013 reported an epilepsy prevalence of 5.4 % among the Finnish Spitz dog population in Finland that were still alive [82]. This epidemiological study provided data regarding phenotype, inheritance and risk factors for idiopathic epilepsy of 141 affected Finnish Spitz dogs. For this study idiopathic epilepsy was defined as at least 2 seizure episodes without interictal neurologic abnormalities; with data collected by questionnaires and telephone interviews [82]. The latter study detected a significant gender predisposition with 60.1 % males and 39.9 % females compared to a control population [82]. The median age of seizure onset was 3 years [82]. The median seizure frequency was 2 seizures per year. A history of cluster seizures was reported for 16.2 % of dogs. The seizure type was defined as focal epileptic seizures in 54 % of the dogs, as focal epileptic seizures evolving into generalised seizures in 31 % of the dogs and as (primary) generalised epileptic seizures in 1 % of the dogs [82]. In 7 % of dogs the seizures were generalised but with unknown onset and in an additional 7 % of dogs the seizure type was unclassified. The median seizure duration was long at 11.75 min (occasionally ≥ 40 min). The disease course was reported as non-progressive in 67.8 % of the dogs and treatment response was assessed as good in 78.9 % of the dogs [82]. The heritability was estimated at 0.22 and, hence, a complex pattern of inheritance such as polygenic recessive or autosomal recessive with incomplete penetrance was suggested [82]. Another study conducted in 2006 focused on EEG and MRI findings in 11 affected Finnish Spitz dogs [81]. Among those dogs the seizure type again predominantly was defined as focal epileptic seizure or as focal epileptic seizure evolving into generalised epileptic seizures (in 73 % of the dogs), with the majority of dogs experiencing the latter seizure type [81]. In 23 % of dogs, episodic behavioural changes were present that lasted for only a few minutes such as disorientation, fear and compulsive walking. These episodes were classified as focal seizure activity, since consciousness was altered during these episodes [81]. Based on the predominant focal seizure type the term focal idiopathic epilepsy was proposed. On EEG examination, focal epileptic activity was found in 64 % of dogs, and generalised epileptic activity was observed in 36 % of dogs [81]. On MRI examination, contrast enhancement was detected within the right parietal cortex in one of the dogs, but was suggested to be a reversible postictal finding, as such changes were not observed in repeated MRI examination of the same dog [81]. The remaining dogs showed no MRI abnormalities [81]. Another EEG study from 2007 – including 15 affected Finnish Spitz dogs – reported that paroxysmal activity seems to originate from a caudal-occipital location [80]. Furthermore, the EEGs of dogs with epilepsy exhibited a significant difference in background frequency bands compared with healthy control dogs; and beyond that, phenobarbital treatment in affected dogs was identified to markedly influence all background activity bands [80]. Recently, a FDG-PET-study has been published that investigated 11 affected Finnish Spitz dogs diagnosed with focal idiopathic epilepsy and six healthy controls [83]. This study identified that epileptic dogs had significantly lower standardized uptake values in numerous cortical regions, the cerebellum, and the hippocampus compared to the control dogs. The lowest standardized uptake values were found in the occipital lobe. Thus, the authors of this study suggested the use of FDG-PET as a diagnostic tool for Finnish Spitz dogs with suspected idiopathic epilepsy [83]. Identified risk factors: A generalised seizure phase was determined to be a risk factor for development of progressive disease [82]. Predisposing factors associated with the occurrence of seizure generalisation were the age of onset (≤3 years), duration of the seizure (1–10 min and ≥ 20 min), number of feeding times per day (only once per day), and when the dog was used for hunting [82].

### Golden Retriever

Three studies focusing specifically on idiopathic epilepsy in Golden Retrievers –all conducted in Switzerland – have been published in the veterinary literature to date [84–86]. These studies provide information about clinical manifestation, heritability and EEG characteristics. Based on the study, 25 affected dogs [86], 36 affected dogs [84] or 5 affected dogs [85] were included, respectively. Prevalence data have not been reported to date, but the Golden Retriever was among the most common affected breeds in an UK epidemiological epilepsy study [17]; however, this may be due to the Golden Retriever being a very popular breed in the UK. Depending on the respective study the mean age at seizure onset ranged between 27.5 months (≈2.3 years) [84] and 24.9 months (≈2 years) [86]. The 1994 study, was a retrospective cohort study, that diagnosed idiopathic epilepsy when normal results on clinical, neurological, laboratory, CSF and EEG examinations were evident [84]. This study found a significant gender predisposition for male dogs (ratio 3.5:1), but only when a distribution of 1:1 for the general dog population was assumed [84]. Generalised epileptic seizures were reported as the most common seizure type among all of the studies with 83 % [86] and 92 % [84], respectively, but a detailed distinction between (primary) generalised epileptic seizures and focal epileptic seizures evolving into generalised epileptic seizures was not provided [84, 86]. Hence, a proportion of dogs may have suffered from focal epileptic seizures evolving into generalised epileptic seizures instead of (primary) generalised epileptic seizures. A long-term treatment study from 1999 included epileptic dogs when they had at least 2 epileptic seizures, normal clinical and neurological examination, normal routine blood work, normal urine analysis and normal CSF analysis. Dogs that were pre-treated with antiepileptic treatment were excluded from the study [86]. The mean seizure frequency was reported with one seizure every 16 days in the latter study [86]. A good initial treatment response to phenobarbital was observed in most of the dogs, however, after 4 years, treatment response was poor with almost half (43 %) of the dogs being euthanized [86]. The mean survival time after diagnosis was 46 months (≈3.8 years) among phenobarbital treated dogs [86]. A continuous positive impact of castration/neutering on seizure course was not found in any of the studies (although in few dogs a transient improvement was initially noted) [84, 86]. An EEG study from 1996 frequently identified spindles in all recordings of five examined epileptic Golden Retrievers [85]. Early data already suggested a genetic base for this breed, based on an increased idiopathic epilepsy prevalence of certain subpopulations and a repeated occurrence in different families of the same sires [84]. Based on pedigree analyses and binomial testing an autosomal multifactorial recessive mode of inheritance was suspected [84]. The identification of a causative gene mutation has not yet been reported. Identified risk factors: Treatment response was better the earlier an antiepileptic treatment was initiated and the lower the pre-treatment seizure frequency [86]. Potential breed-specific diseases that may mimic idiopathic epilepsy: One study that investigated EIC among several dog breeds (Labrador Retrievers and non-Labrador retriever breeds) identified some Golden Retrievers diagnosed with EIC; however, a DNM1-gene mutation was not identified in any of the affected Golden Retrievers [30]. In contrast to epileptic seizures EIC usually is triggered by strenuous exercise and mental status predominantly remains normal during episodes, which may help a clinician to differentiate between both diseases.

### Hungarian (Magyar) Vizsla

To date, one study about clinical characteristics and inheritance of idiopathic epilepsy in Hungarian (Magyar) Vizslas has been published in 2003 [87]. This study was conducted in the United States and summarised information on 29 Hungarian (Magyar) Vizslas diagnosed with idiopathic epilepsy and 114 non-affected siblings and parents [87]. Idiopathic epilepsy was defined on the basis of a dog having 2 or more seizures occurring at least 1 month apart, no evidence of toxin exposure or head trauma, normal serum chemistry results, and a normal neurologic examination. For dogs with an age at seizure onset <6 months or >5 years, unremarkable findings on CT or MRI scans and CSF analysis were required [87]. Five dogs underwent CSF analysis and three dogs had brain-imaging studies [87]. Prevalence data are yet not reported for this breed, but among 1260 dogs with epilepsy in the UK 0.6 % were Hungarian (Magyar) Vizslas [46]. No significant gender predisposition was found with 59 % males compared to 41 % females [87]. The median age of seizure onset was 3 years. The seizure type was defined as focal epileptic seizures in 79 % of dogs and as generalised epileptic seizures in 21 % of dogs. In 22 % of those dogs with focal epileptic seizures, the seizures evolved into generalised epileptic seizures. In other words, 62 % of dogs had focal epileptic seizures, 17 % of dogs had focal epileptic seizures evolving into generalised seizures and 21 % had generalised epileptic seizures. Initial focal epileptic seizure signs consisted of a combination of limb or head tremors, staring, mydriasis, lip smacking, salivating, facial twitching and/or vomiting [87]. Two dogs, diagnosed with focal seizures, exhibited fly-biting episodes which responded to antiepileptic drugs [87]. The median seizure frequency for the study population was 9 seizures per year. Forty-eight per cent of the 29 epileptic dogs received antiepileptic drug treatment with 21 % of those being not well controlled based on their owners subjective opinion [87]. The segregation analysis was consistent with an autosomal recessive inheritance; however, polygenic inheritance could not be excluded [87]. Pedigree analysis revealed that all affected dogs could be traced back to a common sire. The identification of a causative gene mutation has not yet reported [76, 87].

### Irish Wolfhound

In the current literature, one study is available that provides information regarding heritability and clinical characteristics of idiopathic epilepsy in Irish Wolfhounds [24]. The latter study was published in 2006 and conducted in the United States [24]. The diagnosis of idiopathic epilepsy was made based on a history of more than 2 seizures in the absence of other medical problems. Absence of other medical problems was confirmed for all affected dogs by normal physical and neurologic examination results, CBC, serum biochemical analysis, ammonia or bile acid values or both, and urine analysis. Dogs were considered unaffected if no seizure had been observed during the dog’s lifetime. Seizure-free dogs that died before the age of 4 years and dogs who had seizures in the presence of seizure-associated conditions or diseases, compatible with metabolic seizures or structural epilepsy were excluded from the study [24]. Among a population that contained 796 Irish Wolfhounds, 146 dogs with idiopathic epilepsy were identified, leading to an estimated epilepsy prevalence of 18.3 % [24]. In 73 % of dogs the age at seizure onset was under three years old, with males having a later average age at seizure onset than females [24]. The seizure type was predominantly defined as generalised, but a precise description of seizure onset signs (e.g. focal epileptic seizures evolving into generalised seizures) was not provided. A gender predisposition towards males was found (61.6 % affected males versus 38.4 % affected females) compared to the control population [24]. The life expectancy of epileptic individuals compared to the general life expectancy of Irish Wolfhounds (provided by another study [88]) was reduced by two years [24]. The average inbreeding coefficient (calculated throughout 10 generations) for all the dogs entered into the study was 0.156 [24]. The heritability index for the affected dogs, their littermates, and unaffected parents was calculated at 0.87. Pedigree analysis and segregation analysis were best compatible with a complex pattern of inheritance such as an autosomal recessive trait with incomplete penetrance [24]. The identification of a causative gene mutation has not yet been reported. Potential breed-specific diseases that may mimic idiopathic epilepsy: Hyperekplexia (also known as startle disease), which has been described in Irish Wolfhounds is a disease characterized by noise- or touch-induced episodes of muscle stiffness and apnoea [89]. In affected pups clinical signs start 5–7 days post-partum and manifest with handling-evoked extensor rigidity and tremor. A micro-deletion of a presynaptic glycine-transporter-gene (GlyT2, *SLC6A5*) has been identified in affected Irish Wolfhound puppies [89]. Beside startle disease, a genetic predisposition for intrahepatic portosystemic shunts has been reported for the Irish Wolfhound [90]. Therefore potential hepatic encephalopathy needs to be considered in young Irish Wolfhounds that present with seizures [90]. In addition to portosystemic shunts a transient idiopathic hyperammonemia (with normal bile acid testing) due to urea cycle enzyme deficiency has been reported in Irish Wolfhound puppies [91, 92]. However, whether this transient and usually only moderate hyperammonemia may also contribute to seizures has not been investigated in detail, but may be considered in Irish Wolfhounds puppies with seizures and normal bile acid testing.

### Italian Spinone

Very recently, the Italian Spinone was reported to be affected by idiopathic epilepsy [93]. A population study was conducted to estimate the prevalence of idiopathic epilepsy in the Italian Spinoni in the UK and to investigate predictors of survival and seizure remission. The owners of all UK Kennel Club registered Italian Spinoni were invited to complete a phase I questionnaire. The phase I questionnaires, the primary veterinarian’s, and, when available, the veterinary neurologist’s medical records (including results of diagnostic investigations) were reviewed by the investigators to identify Italian Spinoni with idiopathic epilepsy and obtain data on treatment and survival. Additional information on various aspects of epilepsy (including seizure phenomenology and frequency) was obtained from owners of epileptic Italian Spinoni who completed the phase II questionnaire. The phase I questionnaire was returned for 1192 Italian Spinoni, of these, 63 Italian Spinoni were identified with idiopathic epilepsy. The prevalence of idiopathic epilepsy in the Italian Spinoni in the UK was estimated as 5.3 %. Mean age at first seizure was 38 months (median: 35 months). The gender distribution of the epileptic dogs was 67 % males and 33 % females (male to female ratio 2:1), but was not compared to the general Italian Spinone population. The phase II questionnaire was returned for 47 (75 %) of the 63 idiopathic epileptic Italian Spinoni. The most common seizure type was generalised tonic-clonic seizures with impaired consciousness and autonomic manifestations (e.g., increased salivation, urination, defecation) for all 47 Italian Spinoni. Focal epileptic seizures evolving into generalised epileptic seizures were consistently recognized by the owners of 24 Italian Spinoni (51 %). Cluster seizures and status epilepticus occurred anytime in life in 46 (73 %) and 13 (21 %) Italian Spinoni, respectively. Seizure remission occurred in 3 (6 %) of the 47 Italian Spinoni whose owners returned the phase II questionnaire. Successful antiepileptic drug treatment with good seizure control was reported to be challenging in many instances. The identification of a causative gene mutation has not yet reported, but genetic analyses are currently in progress [93]. Identified risk factors: Survival time was significantly shorter in Italian Spinoni euthanised because of poorly controlled epilepsy compared with epileptic Italian Spinoni that died of unrelated disorders. Survival was significantly longer in Italian Spinoni with no cluster seizures and in Italian Spinoni in which antiepileptic medication was initiated after the second seizure rather than after ≥3 seizures [93].

### Labrador Retriever

Three studies investigating idiopathic epilepsy in the Labrador Retriever are currently available; two Swiss [94, 95] and one Danish [26] study. All three studies focus on different aspects of heritability and clinical characteristics. The Swiss studies were cohort studies and idiopathic epilepsy was defined by occurrence of more than one (or two) seizures (depending on study) and normal findings on physical examination, neurological examination, routine blood work, urine and CSF analysis [94, 95]. The two Swiss studies include 54 [94] and 55 [95]. Labrador Retrievers with idiopathic epilepsy, respectively, and they do not provide prevalence data [94, 95]. The Danish study [26] is an epidemiological cross sectional population study, which investigated a reference population of 29,602 Labrador retrievers registered in the Danish Kennel Club in a ten year period. From the reference population a representative sample of 550 dogs were selected for by random sampling stratified by year of birth. After questionnaire interviews of all 550 dog owners and clinical investigation of dogs, suspected with epilepsy, 17 dogs were finally identified with idiopathic epilepsy giving a prevalence of 3.1 % among the investigated Danish population [26]. No gender predisposition or positive effects of castration on epilepsy course was detected in the Swiss or the Danish studies [26, 94, 95]. The Danish study reported the seizure type as generalised epileptic seizures in 24 % of dogs and as focal epileptic seizures or focal epileptic seizures evolving into generalised seizures in 70 % of dogs. Among the latter 70 % of dogs focal epileptic seizures were rare, whereas focal epileptic seizures evolving into generalised seizures were predominant [26]. In both Swiss studies the seizure type was reported to be generalised in almost all the dogs (91 % [94] and 96 % [95], respectively) and only 9 % presented with focal epileptic seizures [94]. But, the latter two Swiss studies did not distinguish between (primary) generalised epileptic seizures and focal epileptic seizures evolving into generalised seizures; and preictal signs were reported in a substantial number of dogs (which were not considered as part/onset of a seizure), which may explain the discrepancy to the seizure type found in the Danish study. Age at seizure onset was reported with a mean age of 30.6 months in one of the Swiss studies [95], with a mean age of 34 months for males and 28 months for females in the other Swiss study [94], and with 76 % of dogs having the first seizure by the age of 4 years in the Danish study [26]. The average seizure frequency in one of the Swiss studies was one every 65 days in dogs with generalised seizures and one every 205 days in dogs with focal seizures, however, approximately half of the dogs had seizures more than once a month [94]. Pedigree analysis was best compatible with a polygenic, recessive inheritance according to one of the Swiss studies [95]. The identification of a causative gene mutation has not yet been reported. Identified risk factors: The 1997 Swiss study found that Labrador Retrievers with a higher age at seizure onset showed a good treatment response, even if treatment began late. Dogs with low seizure frequencies and low total numbers of seizures responded well to therapy if treated as early as possible. Untreated dogs mostly showed a progressive disease course [94]. One study that examined inhibitory and excitatory neurotransmitters in the CSF of epileptic Labrador Retrievers, epileptic non-Labrador Retrievers and non-epileptic control dogs, identified that CSF concentrations of γ-aminobutyric acid (GABA) and glutamate (GLU) were significantly lower in Labrador Retrievers with idiopathic epilepsy than in a control-group of non-epileptic dogs as well as in non-Labrador Retriever dogs with idiopathic epilepsy [96]. However, the GLU to GABA ratio was significantly higher in epileptic Labrador Retriever than in epileptic non–Labrador Retriever dogs. It was suggested that an imbalance between GABAergic inhibition and excitation by glutamate (indicated by the high GLU-GABA ratio) in epileptic Labrador Retrievers may be involved in the epileptogenic processes in this breed [96]. However, whether those findings are the cause or the consequence of seizure activity or a combination of both cannot be concluded and still need to be further elucidated. Potential breed-specific diseases that may mimic idiopathic epilepsy: For the Labrador Retriever EIC, predominantly caused by a DNM1-gene mutation, needs to be considered as potential differentials for epileptic seizures. Several studies suggest the existence of another and “DNM-1-independent” EIC condition in Labrador Retrievers, as some of the EIC-affected Labrador Retrievers are negative or heterozygous for the DNM1-gene mutation (approximately 15–30 % of EIC affected Labrador Retriever) [97, 98]. Hence, two distinct terms have gained acceptance for Labrador Retrievers: d-EIC (homozygous DNM1-gene mutation) and non-d-EIC (negative or heterozygous for the DNM1-gene mutation) [30, 97, 98]. Apart from a suspected diverse genetic background for the latter two EIC types, clinical differences between d-EIC and non-d-EIC have been observed. However, in general, and in contrast to seizures, EIC-episodes are induced by strenuous exercise. Contrary to epileptic seizures muscle tone is initially decreased in the affected limbs and consciousness remains preserved in more than 80 % of the Labrador Retriever with d-EIC. Another study also reported a wide-based pelvic limb stance, crouched posture and falling to the side during d-EIC [99]. For non-d-EIC, the collapse episodes are reported to occur at an older age, furthermore abnormal mentation and involvement of all limbs are observed more frequently^58^. A gene test for d-EIC in Labrador Retrievers is available [30]. Narcolepsy with cataplexy, which may potentially mimic a seizure event, has also been reported in Labrador Retrievers [100, 101]. However, in contrast to seizures a clinical hallmark of narcolepsy, are sudden episodes of muscle atonia triggered by excitement such as presentation of food. Another disease that may mimic seizure episodes in Labrador Retriever puppies is familial reflex myoclonus, in which the affected puppies present with spasticity and opisthotonus at the age of 3 weeks [102], especially when handled or lifted up. In general, the Labrador Retriever breed is not known to be predisposed for NCL, however, one case report exists reporting an eight year old Labrador Retriever diagnosed with NCL on necropsy [103]. This dog had a 11-month history of progressive focal seizures with generalised seizures at the end stage of the disease [103]. Rapid eye movement (REM) sleep disorder is another potential differential when assessing an epileptic Labrador Retriever, with one case report of a 9-month-old Labrador retriever cross presenting with two morphologically distinct types of seizure episodes, one that occurred only during sleep and one that occurred only when awake. An EEG identified that the sleep-associated episodes occurred during REM sleep, consistent with a diagnosis of a REM behaviour disorder, which was improved with a tricyclic antidepressant medication. The waking episodes were diagnosed as epileptic seizures, as there was a clinical response to antiepileptic medication [104].

### Lagotto Romagnolo

To date three studies about clinical signs, heritability and FDG-PET imaging findings in benign familial juvenile epilepsy in the Lagotto Romagnolo dog have been published [105–107]. The first study was published in 2007 and focused on clinical characteristics and clinical course, which both appeared to resemble the human form of benign familial juvenile epilepsy [105]. The latter study included 25 Lagotto Romagnolo puppies with (simple or complex) focal seizures and 3 adult Lagotto Romagnolo dogs exhibiting similar clinical signs [105]. However, prevalence data are yet not available. The mean age of seizure onset is reported as 6.3 weeks of age (with most puppies being affected by 1–2 month of age). The benign disease course is characterized by a spontaneous seizure remission by 8 to 13 weeks of age [105]. The epileptic seizures manifest with episodic stiffness, generalised tremor and a predominantly preserved consciousness. Although this seizure semiology is a rather uncommon presentation for epileptic seizures, epileptiform EEG activity was detected in affected puppies [105]. Puppies that were severely affected also suffered from interictal ataxia and hypermetria. The seizure frequency varied from multiple episodes per day to one episode per week. A gender-predisposition was not found. Histopathologic examination in one puppy and one adult dog, revealed Purkinje cell inclusions and vacuolation of axons restricted to the cerebellum [105]. A simple autosomal recessive inheritance was best compatible with pedigree analysis [105]. Using a genome-wide association study the disease locus was mapped to chromosome 3 where a protein-truncating mutation in the LGI2 gene was identified in 2011 [106]. The latter study showed that LGI2, like the human analogue epilepsy gene LGI1, is neuronally secreted and acts on metalloproteinase-lacking members of the ADAM family of neuronal receptors, which function in synapse remodelling. It was identified that LGI2 truncation, like LGI1 truncations, prevents secretion and ADAM interaction [106]. LGI2 was highly expressed in the immediate post-natal period until halfway through pruning, unlike LGI1, which is expressed in the latter part of pruning and beyond. LGI2 acts at least in part through the same ADAM receptors as LGI1, but earlier, ensuring electrical stability during pruning time, preceding this same function performed by LGI1 later in life [106]. Hence, this functional LGI2-to-LGI1 transition may explain the benign and remitting course of epilepsy in the Lagotto Romagnolo breed. A genetic test is available for Lagotto Romagnolos. One study among a large Lagotto Romagnolo population from 3 different countries identified 32 % dogs as disease carriers [106]. It should be noted that in a small proportion of dogs seizures occurred at an adult age [105]. However, in almost all of these adult cases no LGI2 gene mutation was identified, suggesting that there might exist a second and distinct form of epilepsy in the Lagotto Romagnolo [106]. One recent study focussed on FDG PET-imaging in affected Lagotto Romagnolos [107]. Visual analysis revealed areas of hypometabolism interictally in five out of six dogs with juvenile epilepsy in the occipital, temporal, and parietal cortex. Epileptiform EEG activity occurred in three of these dogs in the same areas where PET showed cortical hypometabolism [107]. Visual analysis showed no abnormalities in cerebral glucose uptake of dogs with adult-onset epilepsy, which further supports the theory of another etiologically different form of genetic epilepsy in this dog breed [107]. Apart from the structural epilepsies that result from neurodegenerative diseases such as progressive myoclonus epilepsy or NCL, the Lagotto Romagnolo is the first dog breed where an idiopathic epilepsy causative gene mutation has been identified. Potential breed-specific diseases that may mimic idiopathic epilepsy: One report describes 2 Lagotto Romagnolo puppies diagnosed with cerebellar cortical abiotrophy, which might be considered as potential differential to benign familial juvenile epilepsy in this dog breed [108]. However, in cerebellar cortical abiotrophy the clinical (cerebellar) signs were reported as rapidly progressive or progressive followed by a static phase with no evidence of an episodic nature [108], which contrasts the episodic signs and remitting disease course evident in benign familial juvenile epilepsy [105]. In one of the cerebellar cortical abiotrophy affected puppies the cerebellum was slightly decreased in size on MRI examination of the brain [108].

### Petit Basset Griffon Vendeen (PBGV)

One retrospective epidemiological population study investigating clinical characteristics and prevalence of epilepsy in PBGV dogs has been published in 2011 (including both living and deceased dogs) [25]. This study was conducted in Denmark and included all PBGV dogs (=820) registered in the Danish Kennel Club between 1999–2008. Idiopathic epilepsy was defined on the basis of a dog having at least 2 seizures with a minimum interval of 24 h and the dog having a typical epileptic seizure phenomenology [25]. All dogs that were defined as epilepsy positive after an interview validation and that were still alive, were invited to participate in a clinical evaluation at the study centre, which included clinical examination, neurological examination and blood samples analysed by CBC, serum biochemistry including thyroid hormone concentration and urinalysis, ECG and ultrasound of the heart by a cardiologist (*n* = 19). A few owners were offered an MRI of their dog’s brain (*n* = 3) [25]. Forty-two dogs were evaluated to be true epilepsy cases and the epilepsy prevalence among the Danish PBGV population was estimated at 8.9 % [25]. The median age at seizure onset was 2 years. The gender distribution was 62 % males and 38 % females, but there was no significant gender predisposition detected (compared to the general PBGV population) [25]. The seizure type was defined as focal epileptic seizures in 41 % of dogs and as focal epileptic seizures evolving into generalised epileptic seizures in 52 % of dogs. In 5 % of dogs generalised epileptic seizures were identified and in 2 % of dogs the seizures remained unclassified. The most commonly reported focal seizure signs included motor signs such as ataxia and contractions of single muscle groups, autonomic signs such as vomiting and salivation and paroxysms of behavioural signs such as excessive attention seeking or standing with a blank stare not responding to external stimuli. The typical seizure duration varied from one to three minutes. A strong litter effect was demonstrated supporting the hypothesis of a hereditary component of epilepsy in the PBGV. Identification of a causative gene mutation has not been reported [25].

### Shetland Sheepdog

Three reports about epilepsy in Shetland Sheepdogs are available in the current literature [109–111]. All studies were conducted in Japan. The first study was published in 2002 and reports about a large family of Shetland Sheepdogs with natural occurring familial frontal lobe epilepsy defined by EEG analysis and seizure semiology [109]. Two litters of one large family were produced deliberately for prospective examination in this study. A detailed definition for idiopathic epilepsy was not provided [109]. The age at seizure onset was predominantly between 1 and 1.5 years of age. The average seizure frequency varied from one seizure every week to one every 6 months. The gender distribution was 79 % females compared to 21 % males. The seizure type was predominantly defined as generalised in almost all cases, but detailed classification of initial seizures signs was not conducted [109], hence a proportion of dogs might have experienced focal seizures evolving into generalised seizures instead of (primary) generalised seizures. EEG examination identified paroxysmal discharges predominantly in the frontal lobes [109]. Based on this, this epilepsy was postulated as familial frontal lobe epilepsy, however with prolonged disease duration also the parietal, temporal and occipital lobes showed epileptiform activity on EEG [109]. Pedigree analysis excluded potential mitochondrial or sex-linked inheritance and a multifactorial inheritance was suggested to be most likely [109]. Identification of a causative gene mutation has not yet reported. Additional findings of the latter study were increased aspartate and glutamate levels in the CSF in some of the epileptic dogs compared to control dogs [109]. Hence, another study that was published in 2005 focussed on intracerebral microdialysis and EEG recording as well as histopathological examination of epileptic Shetland Sheepdogs [110]. Intracerebral microdialysis and EEG – both conducted during hyperventilation – revealed increased extracellular glutamate and aspartate concentrations in the cerebral cortex of epileptic dogs. Coinciding with the increase in excitatory neurotransmitters, an increase in paroxysmal discharges on EEG was detected. On histopathological examination, dogs affected by status epilepticus showed a reduced density of glutamate receptors in the area of the lateral nucleus of the thalamus. In addition, glutamate positive granules were found within the perineural spaces of the cerebral cortex. It was considered possible that a decrease of glutamate receptor levels may induce an increase in extracellular glutamate concentration, which would evoke neuronal hyperexcitability and may contribute to a collapse of extracellular glutamate regulation during status epilepticus [110]. Another case report of a Shetland sheepdog with drug resistant epilepsy identified hippocampal and mesial temporal lobe sclerosis on necropsy. However, this finding was suggested as a secondary phenomenon induced by recurrent seizures rather than to be a primary seizure-causing finding [111]. Potential breed-specific diseases that may mimic idiopathic epilepsy: For the Shetland sheepdog a spongiform encephalopathy has been reported, which appears with neurological signs that may mimic a seizure event. However, clinical manifestation is early within the first weeks of life (between the 2– 9 weeks of life) and consists of tremors, ataxia, paresis, spasticity and loss of cranial nerve function. DNA sequencing of affected puppies showed a point mutation that resulted in an amino acid change of mitochondrial encoded cytochrome b [112]. The Shetland sheepdog is also a dog breed frequently affected by ABCB1/MDR1-gene mutation, with identified mutant allele frequencies between 1 − 12 % depending on the respective study and geographic area [33], which may need to be considered in Shetland sheepdogs with acute seizures and potential previous exposure to neurotoxic P-gp substrates.

### Standard Poodle

In the current literature, there are two studies available that provide information about idiopathic epilepsy in the Standard Poodle [65, 113]. One study, that was published in 2007, reports clinical characteristics and mode of inheritance of epilepsy in a large family of Standard Poodles in the United States [113]. This study included 30 Standard Poodles diagnosed with probable idiopathic epilepsy and 90 healthy controls. Dogs were defined as having’probable idiopathic epilepsy’ if they had had at least 1 seizure without any evidence of an underlying cause. Specifically, the following conditions all had to be met: the owner’s answers to health-related questions revealed no illnesses or events (e.g., head trauma) that could plausibly account for the seizures, at least 1 year had passed since seizure onset during which no interictal neurologic abnormalities were observed, and the dog was between 6 months and 7.5 years old when seizures began [113]. The term’probable’ was used because the medical work up was insufficient to definitely exclude other causes of epilepsy and because dogs that had experienced only a single seizure were also included [113]. No significant gender predisposition was detected between affected males (57 %) and females (43 %). The median age at seizure onset was 3.7 years, however, 20 % of all affected dogs had their first seizure after an age of five years [113]. The seizure type could be determined in 29 dogs and was defined as focal epileptic seizures in 33 % and as focal epileptic seizure evolving into generalised epileptic seizures in 60 % of dogs [113]. Overall 93 % of the dogs had focal epileptic seizures or focal epileptic seizures evolving into generalised seizures. In 3.5 % of dogs seizures were classified as (primary) generalised epileptic seizures; in another 3.5 % of dogs the epileptic seizures were generalised but the exact onset of seizures could not be determined precisely [113]. As the majority of dogs (93 %) among this family experienced focal epileptic seizures or focal epileptic seizures evolving into generalised seizures, familial focal epilepsy was suggested [113]. Focal epileptic seizures consisted of shaking, jerking, or shivering; incoordination characterized by staggering or an inability to stand; stiffness or rigidity; and unusual movements or body positions such as a head tilt or awkward limb lifting. Autonomic signs included hypersalivation, panting, urination, and increased heart rate. Some dogs also presented with increased anxiety or automatisms such as licking, lip smacking, swallowing or circling [113]. Only 13 % of the dogs received antiepileptic drug treatment and all of them showed a good treatment response. The segregation analyses suggested a recessive autosomal inheritance with almost complete penetrance [113]. All examined Standard poodles in the latter study were closely related; hence, it is possible that there might be different epilepsy courses and modes of inheritance in geographically and genetically distinct standard poodle populations. However, a frequent occurrence of focal or focal epileptic seizures evolving into generalised seizures in Standard poodles was already reported in an earlier study from 2002 [65]; but the two studies (2002 and 2007) were conducted by the same authors and it was mentioned that few of the dogs were included in both studies. The study from 2002 reported a median age at onset of 2.4 years, a median seizure frequency of 2.8 seizures per year and a history of cluster seizures in 34.1 % of affected Standard Poodles [65]. Identification of a causative gene mutation has not yet been reported [65, 113]. Potential breed-specific diseases that may mimic idiopathic epilepsy: For the Standard Poodle, a neonatal encephalopathy with seizures (NEWS) has been reported that may play a role as potential differential in Standard poodle puppies with seizures [114]. However, NEWS manifests immediately after birth with ataxia, tremors and generalised tonic-clonic seizures. As a rule, affected puppies die within the first two months of life. A missense mutation in the canine orthologue of ATF2 has been identified in affected puppies [114]. Polymicrogyria is a rare malformation of the cerebrum characterized by an excessive number of small, histologically anomalous gyri and has been described in Standard Poodles [115, 116]. Affected dogs experience cortical blindness and other neurologic abnormalities including abnormal (hypermetria) gait and behavioural changes [115, 116] and may be considered as differential in young Standard Poodles that present with focal seizures or behavioural changes. Neurological signs predominantly start at a very young age (<4 months). MRI examination is consistent with multiple disorganized gyri, which especially may be seen on T2-weighted dorsal plane images [115]. EEG-examination of 1 dog revealed epileptiform discharges, including both spike and spike and wave discharges with voltage maximum potentials over the parietal/occipital region, which supported the repetitive behaviour as focal seizures [115].

### German Shepherd, Beagle, Dachshund and Keeshond

There are a few older publications available that specifically focus on epilepsy in German Shepherds, Beagles, Dachshunds or Keeshonds [117–120]. Particularly for the Beagle, it should be mentioned that most of the earlier published data are based mainly on laboratory dog populations [117]. However, one recent epidemiological investigation and one genetic investigation support an increased risk for idiopathic epilepsy in Beagles at present [22, 76]. In Beagles, Dachshunds (miniature wirehaired) and Basset Hounds it is important to consider the occurrence of progressive myoclonus epilepsy (Lafora disease), as the latter is considered a neurodegenerative disorder and structural-metabolic epilepsy rather than idiopathic epilepsy [121–123]. In Dachshunds (and Bassett Hounds) a gene test for progressive myoclonus (Lafora) epilepsy is available [124]. Data from the Dachshund Breed council surveys in 2012 and 2015 suggest a epilepsy prevalence of approximately 1 % but rising to 3.7 % in miniature long haired Dachshunds (personal communication Clare Rusbridge February 2015). For the Keeshond, older studies proved a clear founder effect, which was most likely consistent with an autosomal recessive inheritance [120, 125], the median age at onset was reported with 2 years and some EEG examination has been made, however, further detailed clinical data are lacking. There are no up-to date studies available about idiopathic epilepsy in German Shepherds Dogs, but several current epidemiological canine epilepsy studies have been published that include interesting information for this breed. Most of those epidemiological studies have been conducted in the UK, and most of them identified the German Shepherd Dog among the most common epilepsy affected breeds [17, 46, 47]. Another epidemiological study revealed the German Shepherd Dog has an increased risk of cluster seizures compared to other breeds like the Labrador Retriever [126].

Finally, it is important to consider that in addition to the above mentioned dog breeds, there is strong clinical evidence that many more purebreds, such as the Siberian Husky, Staffordshire Bull Terrier, Boxer dog, Greater Swiss Mountain dog, Schipperke and many others appear to be affected by idiopathic epilepsy [10, 17, 22, 46, 47, 76]. It is only a matter of time until detailed data regarding the clinical characteristics and inheritance for those so far “suspected” breeds will be published. Furthermore, current epidemiological data suggest that beside purebred dogs, crossbreeds with idiopathic epilepsy present an increasing proportion among canine idiopathic epilepsy populations [46].

## Conclusion and future perspectives

The present manuscript was conceived to review current knowledge of idiopathic epilepsy in purebreds with special interest on breed-specific phenotypes including clinical characteristics, disease course, seizure control and genetic transmission. Some differences, and in parts even contradicting findings became evident among breeds, and even – at least to some degree – between geographically distinct populations of the same breed. This may to some degree reflect the development over time in our understanding of epilepsy, different study designs (see Table 4) and definitions for epilepsy and seizure terms that have been applied and furthermore genetic diversity between dog breeds and in some cases between geographically distinct populations of the same breed. Frequently, variable study designs were evident, with individual studies containing variable levels of disease-specific parameters (e.g. analysing occurrence of cluster seizures or not) and moreover data collections were performed in different ways (e.g. prospectively versus retrospectively). In human medicine, general guidelines and classification systems have long been established under the care of the International League Against Epilepsy. As these guidelines remain constantly under progress and are kept updated over the years, they have substantially promoted research and care for epilepsy patients, especially by diagnosis and treatment [127–133]. In recent years, strong efforts have been made to classify and define epilepsy terms in veterinary medicine, in particular, the clinical classification of different seizure types has been advanced by the application of human medicine classification systems [2, 65, 134–137]. However, direct comparability between canine epilepsy studies remains limited to some degree, due to the above mentioned issues, which point once more to the need for generally accepted concepts and guidelines for the conduct of epilepsy studies among the veterinary community, under guidance of a specialised veterinary epilepsy task force. The currently formed International Veterinary Epilepsy Task Force has crafted several consensus statements to help overcome these challenges in the area of Classification, Diagnosis, Treatment, Neuroimaging, Treatment outcomes, and Neuropathology. This might help establish new consistent studies of breed-specific canine epilepsy phenotypes and syndromes, which in turn may help to promote genetic analysis and to establish novel antiepileptic drug treatment strategies.

**Table 4:** Depicting variable study design

Breed	Study	N	Study designs	Case selection	Inclusion criteria	Exclusion criteria	Investigations for confirmation of idiopathic epilepsy
Australian Shepherd	Weissl et al. 2012 [9]	50	Cohort, controls	Questionaire &phone interview	≥2 seizures ≥ 4 weeks apart, age at onset ≤ 5 years	History of skull trauma	PE, NE, laboratory with bile acid stimulation test
							MRI/CSF (47 %)
							Urinary organic/amino acids (20 %)
							Post mortem (4 %)
Belgian Shepherd	Berendt et al. 2008 [23]	49	Population survey (breed)	Questionnnaire (validated) &phone interview	≥2 seizures	n.s.	n.s.
	Seppala et al. 2012 [34]	94	Cohort, controls	Questionnaire	≥2 seizures	n.s.	Detailed examination (18 %) [35]
							PE, NE, laboratory
							MRI/CSF
							Descriptive: EEG (18 %)^b^
	Oberbauer et al. 2003 [35]	164	Cohort (family-based)	Owner-reported generalized seizures & questionnaire	≥2 seizures	n.s.	n.s.
	Oberbauer et al. 2010 [36]	74	Cohort, controls	Owner and veterinarian reported generalized seizures	≥2 seizures	n.s.	n.s.
	Gullov et al. 2012 [37]	51	Cohort, controls (family-based)	Questionnaire & phone interview	≥2 seizures	n.s.	PE, NE, laboratory with thyroid profile, ECG
	Berendt et al. 2009 [41]	66	Cohort (family-based)	Questionnaire & phone interview	≥2 seizures	History suggesting intracranial disease and progressive neurological signs.	PE, NE, laboratory with thyroid profile, ECG
	Famula et al. 1997 [38]	23	Population survey (breed)	Questionnaire	1 seizure	n.s.	n.s.
		142			≥2 seizures		
	Famula & Oberbauer 2000 [39]	21	Population survey (breed)	Questionnaire	1 seizure	n.s.	n.s.
		157			≥2 seizures		
Bernese Mountain Dog	Kathmann et al. 1999 [45]	50	Cohort	Questionnaire	History of epileptic seizures	n.s.	PE, NE, laboratory with bile acid stimulation test, CSF
Border Collie	Hülsmeyer et al. 2010 [8]	49	Cohort	Questionnaire & phone interview	≥2 seizures, at least 4 weeks apart	Presence of any initial precipitating event (eg, head trauma), an identified brain lesion, or observational data consisting of less than 10 h/day.	PE, NE, laboratory
Border Terrier	Kearsley-Fleet et al. 2013 [17]	n.s.	Population survey (vet practice)	Electronic patient records	≥2 seizures for ≥ 1 year, or	Medical records documented disease that could have caused epilepsy including brain imaging abnormalities	PE, laboratory
					≥4 prescript- ions of AEDs		
	Kloene et al. 2008 [56]	47	Population survey (breed)	Questionnaire	n.s.	n.s.	PE, NE, laboratory with bile acid stimulation test^a^ (10 %)
							CT/CSF (10 %)
							Urinary organic/amino acids (47 %)
Cavalier King Charles	Rusbridge&Knowler 2004 [58]	40	Cohort, controls (family investigation)	Owner-reported seizures	Diagnosis by veterinarian (generalized seizures, AED)	Clinical signs of CM	n.s.
	Driver et al. 2013 [59]	29	Cohort, controls	Medical record search CKCS with CM	History of epileptic seizures	MR lesions other than CM or ventriculomegaly	Laboratory with bile acid stimulation test, CSF, EEG^b^
						Abnormal laboratory data	
Collie	Munana et al. 2012 [64]	29	Cohort	Questionnaire	Age of onset > 6 m/< 5 y, > 6 m prescription of AEDs		Laboratory
Dalmatian	Licht et al. 2002 [65]	11	Cohort	Questionnaire & phone interview	≥1 seizure	Evidence for structural epilepsy, seizures were not seen from the beginning	PE, NE, laboratory, bile acid stimulation test, tests for suspected toxin exposure
English Springer Spaniel	Patterson et al. 2005 [74]	45	Cohort	Questionnaire & phone interview	≥2 seizures, ≥ 4 weeks apart	Evidence for head trauma or toxin exposure	NE, laboratory age of onset < 6 m />5 y: MRI or CT WNL or > 2 y without interictal abnormalities
	Kearsley-Fleet et al. 2013 [17]	n.s.	Population survey (vet practice)	Electronic patient records	≥2 seizures for ≥ 1 year, or	Medical records documented disease that could have caused epilepsy including brain imaging abnormalities	PE, CBC, biochemical profile
					≥4 prescriptions of AEDs		
Finnish Spitz	Jeserevic et al. 2007 [80]	15	Cohort, controls		≥2 focal seizures		PE, NE, laboratory
							MRI/CSF (73 %), EEG^b^
	Viitmaa et al. 2006 [81]	11	Cohort, controls		≥2 focal seizures	Evidence for structural epilepsy	PE, NE, laboratory, MRI/CSF, EEG^b^
	Viitmaa et al. 2013 [82]	111	Population survey (breed)	Questionnaire & phone interview	≥2 seizure episodes	Interictal neurologic abnormalities, onset < 1 y, only 1 seizure episode	PE, NE, laboratory (27.8 %)
	Viitmaa et al. 2014 [83]	11	Cohort		≥2 focal seizures		PE, NE, laboratory
Golden Retriever	Srenk&Jaggy 1996 [85]	5	Cohort, controls	Questionnaire, medical records review	History of epileptic seizures, normal diagnostic tests	n.s.	EEG^b^
	Srenk et al. 1994 [84]	36	Cohort	Questionnaire, medical records review	History of epileptic seizures, normal diagnostic tests	n.s.	PE, NE repeatedly, laboratory with bile acids or ammonia, CSF, EEG
	Lengweiler&Jaggy 1999 [86]	25	Cohort	Questionnaire	≥2 seizures	n.s.	PE, NE repeatedly, laboratory with bile acids or ammonia, CSF
Hungarian (Magyar) Vizsla	Patterson et al. 2003 [87]	29	Population survey (breed)	Questionnaire	≥2 seizures, ≥ 1 month apart	Evidence of toxin exposure or head trauma	NE, laboratory
							If < 6 m/ > 5 < y: CT /MRI/CSF
Irish Wolfhound	Casal et al. 2006 [24]	146	Population survey (families)	Questionnaire	≥2 seizures	Other medical problems	PE, NE, laboratory with bile acids or ammonia or both post mortem exam (10 %)
Italian Spinone	DeRisio et al. 2015 [93]	63	Population survey (breed)	Questionnaire	≥2 seizures, ≥ 24 h apart	n.s.	PE, NE, laboratory
					onset >6 m / <6y		
Labrador Retriever	Heynold et al. 1997 [94]	54	Cohort (medical records)	Questionnaire	≥2 seizures video documentation (28 %)		PE, NE, laboratory with bile acids or ammonia, CSF
	Jaggy et al. 1998 [95]	55	Cohort, controls	Questionnaire	≥2 seizures	Other medical problems	PE, NE, laboratory with bile acids or ammonia, CSF at presentation and at 6 month follow-up
Lagotto Romagnolo	Jokinen et al. 2007 [105]	25	Cohort, case–control	Breeder-reported seizures families	Seizure episodes	n.s.	PE, NE, laboratory, MRI/CSF
							EEG/EMG/BAER^b^
							Post mortem (*n* = 1)
Petit Basset Griffon	Gullov et al. 2011 [25]	42	Population survey (breed)	Questionnaire (validated) & phone interview%	≥2 seizures, ≥ 24 h apart	n.s.	Laboratory, thyroid function, cardiac exam (45 %) bile acid stimulation test (21 %)
							MRI (7 %)
Shetland Sheepdog	Morita et al. 2002 [109]	11	Cohort (family)	Prospective investigation	Repeated seizures	n.s.	Laboratory
							EEG on repeated occasions^b^
							CSF (64 %)
							Post mortem (64 %)
Standard Poodle	Licht et al. 2007 [113]	30	Population survey (family)	Short questionnaire, & phone interview, 6 months follow-up	≥1 seizure	History of illness or head trauma that could account for seizures	≥1 year unremarkable follow-up
						Age of onset <6 m/>7.5y	
	Licht et al. 2002 [65]	41	Cohort	Owner-reported seizures	≥1 seizure	Evidence for structural epilepsy, seizures were not seen from the beginning	PE, NE, laboratory, bile acid stimulation test, tests for suspected toxin exposure
				Questionnaire & phone interview			

^a^some dogs with elevated bile acids; ^b^ EEG was used for descriptive purposes and not as a diagnostic test for IE

*n.s.* not specified, *PE* physical examination, *NE* neurological examination, *MRI* magnetic resonance imaging, *CSF* cerebrospinal fluid analysis, *EEG* electroencephalography, *ECG* electrocardiogram

## Abbreviations

ABCB1: ATP-binding cassette sub-family B
AED: Antiepileptic drug
CBC: Complete blood count
CECS: Canine epileptoid cramping syndrome
CSF: cerebrospinal fluid
CT: Computed tomography
DNM1: dynamin-1
ECG: Electrocardiogram
EEG: Electroencephalography
FDG: Fluorodeoxyglucose
GABA: Gamma-aminobutyric acid
GLU: Glutamate
MDR1: Multi-drug resistance protein 1
NCL: Neuronal ceroid lipofuscinosis
NEWS: Neonatal encephalopathy with seizures
REM: Rapid eye movement
PBGV: Petit Basset Griffon Vendeen
PET: Positron emission tomography
Pgp: P-glycoprotein EIC Exercise induced collapse, UK United Kingdome, DNA Deoxyribonucleic acid, LGI Leucine-rich glioma inactivated
